# Supplementary material for: Inflammation impairs post-hospital discharge growth among children hospitalised with acute illness in sub-Saharan Africa and south Asia
Source: Nat Commun. 2025 Nov 28;16:10788. doi: 10.1038/s41467-025-66245-2 (PMC12663110; doi:10.1038/s41467-025-66245-2)
Supplement: Supplementary file 1 — Supplementary information [file 41467_2025_66245_MOESM1_ESM.pdf]

Title: Inflammation impairs post-hospital discharge growth among children hospitalised with acute illness in sub-Saharan Africa and south Asia.

Authors: James M. Njunge[1,2,*], Evans O. Mudibo[1,2,3], Jasper Bogaert[4], Benedict Orindi[2], Charles J. Sande[2], Celine Bourdon[5], Caroline Tigoi[1,2], Moses M. Ngari[1,2], Narshion Ngao[1,2], Elisha Omer[1,2], Wilson Gumbi[1,2], Robert Musyimi[1,2], Molline Timbwa[1,2], Shalton Mwaringa[1,2], Agnes Gwela[1,2], Johnstone Thitiri[1,2], Ezekiel Mupere[6], Christina L. Lancioni[7], Gerard Bryan Gonzales[3,8], Benson O. Singa[1,9], Emmie Mbale[10], Wieger P. Voskuijl[10,11,12], Donna M. Denno[13,14], Abdoulaye Hama Diallo[15], Roseline Maïmouna Bamouni[15], Mohammod Jobayer Chisti[16], Abu Sadat Mohammad Sayeem Bin Shahid[16], Tahmeed Ahmed[16], Ali Faisal Saleem[17], Syed Asad Ali[17], Holm H. Uhlig[18], Kirkby D. Tickell[14], Robert H.J. Bandsma[5,19], Judd L. Walson[1,20], James A. Berkley[1,2,21]

Affiliations:

1. The Childhood Acute Illness and Nutrition Network; Nairobi, Kenya.
2. KEMRI-Wellcome Trust Research Programme; Kilifi, Kenya.
3. Division of Human Nutrition and Health, Wageningen University and Research; Wageningen, Netherlands.
4. Department of Data Analysis, Faculty of Psychology and Educational Sciences, Ghent University; Ghent, Belgium.
5. Centre for Global Child Health, The Hospital for Sick Children; Toronto, Ontario, Canada.
6. Department of Paediatrics and Child Health, Makerere University College of Health Sciences; Kampala, Uganda.
7. Department of Paediatrics, Oregon Health and Science University; Portland, OR, USA.
8. Department of Public Health and Primary Care, Faculty of Medicine and Health Sciences, Ghent University, Ghent, Belgium.
9. Center for Clinical Research, Kenya Medical Research Institute; Nairobi, Kenya.
10. Department of Paediatrics and Child Health, Kamuzu University of Health Sciences, Blantyre, Malawi.
11. Amsterdam UMC location University of Amsterdam, Amsterdam Centre for Global Child Health, Emma Children's hospital, Amsterdam University Medical Centres, Amsterdam, the Netherlands
12. Amsterdam UMC location University of Amsterdam, Department of Global Health, Amsterdam Institute for Global Health and Development, Amsterdam University Medical Centres, Amsterdam, the Netherlands
13. Department of Paediatrics, University of Washington, Seattle, Washington, USA
14. Department of Global Health, University of Washington, Seattle, Washington, USA
15. Department of Public Health, Faculty of Health Sciences, University of Ouagadougou; Ouagadougou, Burkina Faso.
16. Nutrition Research Division, International Centre for Diarrhoeal Disease Research; Bangladesh (icddr,b), Dhaka 1212, Bangladesh.
17. Department of Paediatrics and Child Health, Aga Khan University Hospital; Karachi, Karachi, Pakistan.
18. Translational Gastroenterology Unit, John Radcliffe Hospital, University of Oxford; Oxford, UK.
19. Department of Biomedical Sciences, University of Malawi College of Medicine; Blantyre, Malawi.
20. Departments of International Health, Medicine and Pediatrics, Johns Hopkins University; Baltimore, Maryland, USA.
21. Center for Tropical Medicine and Global Health, University of Oxford; Oxford, UK.

* Corresponding author: jnjunge@kemri-wellcome.org

Supplementary Methods

Table S 1: ***Selection of systemic inflammation and growth mediator proteins from the SomaScan assay****. Proteins from the SomaScan assay classified by the UniProt Knowledgebase (UniProtKB), as inflammatory response and/or innate immunity from the SomaScan assay were binned into one group we termed systemic inflammation and comprised of 338 proteins. Additionally, proteins classified by UniProtKB as Growth arrest, Growth factor, Growth factor binding, Growth factor receptor, Hormones, Obesity, Osteogenesis and Chondrogenesis were binned into a second group termed growth mediators (GM) and comprised of 297 proteins.*

Biomarkers	UniprotKB protein group key word	UniprotKB proteins (n)	SomaScan proteins (n)
**Systemic Inflammation**	Inflammatory response	186	128
	Innate immunity	396	210
**Growth mediators**	Growth arrest	19	7
	Growth factor	132	116
	Growth factor binding	19	13
	Growth factor receptor	59	32
	Hormones	93	68
	Obesity	65	21
	Osteogenesis	38	25
	Chondrogenesis	17	15

Table S 2: Selected plasma proteins as biomarkers for intestinal inflammation and permeability.

**Protein name**	**Gene**	**UniProt**	**Entrez Gene Symbol**	**Comments on functions and tissue specificity as detailed in UniprotKB (https://www.uniprot.org/ retrieved on 27042025**
Fatty acid-binding protein, intestinal	*FABP2*	P12104	*FABP2*	FABPs are thought to play a role in the intracellular transport of long-chain fatty acids and their acyl-CoA esters. *FABP2* is probably involved in triglyceride-rich lipoprotein synthesis. Binds saturated long-chain fatty acids with a high affinity, but binds with a lower affinity to unsaturated long-chain fatty acids. *FABP2* may also help maintain energy homeostasis by functioning as a lipid sensor. Expressed in the small intestine and at much lower levels in the large intestine. Highest expression levels in the jejunum. PubMed:14563446
Tight junction protein ZO-1	*ZO1*	Q07157	*TJP1*	*TJP1*, *TJP2*, and *TJP3* are closely related scaffolding proteins that link tight junction (TJ) transmembrane proteins such as claudins, junctional adhesion molecules, and occludin to the actin cytoskeleton (PubMed:7798316, PubMed:9792688). Forms a multistranded *TJP1*/*ZO1* condensate which elongates to form a tight junction belt, the belt is anchored at the apical cell membrane via interaction with *PATJ* (By similarity). The tight junction acts to limit movement of substances through the paracellular space and as a boundary between the compositionally distinct apical and basolateral plasma membrane domains of epithelial and endothelial cells. Necessary for lumenogenesis, and particularly efficient epithelial polarization and barrier formation (By similarity). Plays a role in the regulation of cell migration by targeting CDC42BPB to the leading edge of migrating cells (PubMed:21240187). Plays an important role in podosome formation and associated function, thus regulating cell adhesion and matrix remodeling (PubMed: 20930113). With *TJP2* and *TJP3*, participates in the junctional retention and stability of the transcription factor DBPA, but is not involved in its shuttling to the nucleus (By similarity). May play a role in mediating cell morphology changes during ameloblast differentiation via its role in tight junctions (By similarity). The alpha-containing isoform is found in most epithelial cell junctions. The short isoform is found both in endothelial cells and the highly specialized epithelial junctions of renal glomeruli and Sertoli cells of the seminiferous tubules. Further refs PubMed:(31473225, 7798316, 20930113)
Occludin	*OCLN*	Q16625	*OCLN*	May play a role in the formation and regulation of the tight junction (TJ) paracellular permeability barrier. It is able to induce adhesion when expressed in cells lacking tight junctions. PubMed:19114660. Localized at tight junctions of both epithelial and endothelial cells. Highly expressed in kidney. Not detected in testis. PubMed:23239027

Claudin-1	*CLD1*	O95832	*CLDN1*	Claudins function as major constituents of the tight junction complexes that regulate the permeability of epithelia. While some claudin family members play essential roles in the formation of impermeable barriers, others mediate the permeability to ions and small molecules. Often, several claudin family members are coexpressed and interact with each other, and this determines the overall permeability. *CLDN1* is required to prevent the paracellular diffusion of small molecules through tight junctions in the epidermis and is required for the normal barrier function of the skin. Required for normal water homeostasis and to prevent excessive water loss through the skin, probably via an indirect effect on the expression levels of other proteins, since *CLDN1* itself seems to be dispensable for water barrier formation in keratinocyte tight junctions (PubMed: 23407391). Strongly expressed in liver and kidney. Expressed in heart, brain, spleen, lung and testis. PMID: 9931503
Cadherin-1	*Cadherin E*	P12830	*CDH1*	Cadherins are calcium-dependent cell adhesion proteins (PubMed:11976333). They preferentially interact with themselves in a homophilic manner in connecting cells; cadherins may thus contribute to the sorting of heterogeneous cell types. *CDH1* is involved in mechanisms regulating cell-cell adhesions, mobility and proliferation of epithelial cells (PubMed:11976333). Promotes organization of radial actin fiber structure and cellular response to contractile forces, via its interaction with *AMOTL2* which facilitates anchoring of radial actin fibers to CDH1 junction complexes at the cell membrane (By similarity). Plays a role in the early stages of desmosome cell-cell junction formation via facilitating the recruitment of *DSG2* and *DSP* to desmosome plaques (PubMed:29999492).
Junctional adhesion molecule A	*JAM-A*	Q9Y624	*F11R*	Seems to play a role in epithelial tight junction formation. Appears early in primordial forms of cell junctions and recruits PARD3 (PubMed:11489913). The association of the PARD6-PARD3 complex may prevent the interaction of PARD3 with *JAM1*, thereby preventing tight junction assembly (By similarity). Plays a role in regulating monocyte transmigration involved in integrity of epithelial barrier (By similarity). Ligand for integrin alpha-L/beta-2 involved in memory T-cell and neutrophil transmigration (PubMed:11812992). Expressed in endothelium, epithelium and leukocytes (at protein level).
D-amino-acid oxidase	*OXDA*	P14920	*DAO*	Catalyzes the oxidative deamination of D-amino acids with broad substrate specificity. Required to catabolize D-amino acids synthesized endogenously, of gastrointestinal bacterial origin or obtained from the diet, and to use these as nutrients (By similarity). Regulates the level of D-amino acid neurotransmitters in the brain, such as D-serine, a co-agonist of N-methyl D-aspartate (NMDA) receptors, and may modulate synaptic transmission (PubMed: 17303072). Catalyzes the first step of the racemization of D-DOPA to L-DOPA, for possible use in an alternative dopamine biosynthesis pathway (PubMed:17303072). Also catalyzes the first step of the chiral inversion of N(gamma)-nitro-D-arginine methyl ester (D-NNA) to its L-enantiomer L-

				NNA that acts as a nitric oxide synthase inhibitor (By similarity). The hydrogen peroxide produced in the reaction provides protection against microbial infection; it contributes to the oxidative killing activity of phagocytic leukocytes and protects against bacterial colonization of the small intestine (By similarity). Enzyme secreted into the lumen of the intestine may not be catalytically active and could instead be proteolytically cleaved into peptides with antimicrobial activity (By similarity). The hydrogen peroxide produced in the reaction may also play a role in promoting cellular senescence in response to DNA damage (PubMed:30659069). Could act as a detoxifying agent which removes D-amino acids accumulated during aging (PubMed:17303072). Expressed in the cerebellum, in astrocytes of the cortex, in motor neurons and fibers of the lumbar spinal cord (at protein level) (PubMed: 17880399, PubMed: 18544534, PubMed: 18560437, PubMed: 24138986, PubMed: 34041270). Expressed in goblet cells of the small intestine (at protein level) (PubMed:27670111). Increased in the cerebellum of schizophrenic patients (at protein level) (PubMed:17880399, PubMed: 18560437). Decreased in motor neurons of the spinal cord of patients with amyotrophic lateral sclerosis (at protein level) (PubMed:24138986). Expressed in the cerebellum, spinal cord, kidney, and thalamus (PubMed:17880399). Abundant in glia of the cerebellum and predominantly neuronal in the dorsolateral prefrontal cortex, hippocampus and substantia nigra (PubMed:17880399).
Desmoglein-3	*Desmoglein-3*	P32926	*DSG3*	A component of desmosome cell-cell junctions which are required for positive regulation of cellular adhesion (PubMed: 31835537). Required for adherens and desmosome junction assembly in response to mechanical force in keratinocytes (PubMed: 31835537). Required for desmosome-mediated cell-cell adhesion of cells surrounding the telogen hair club and the basal layer of the outer root sheath epithelium, consequently is essential for the anchoring of telogen hairs in the hair follicle (PubMed:9701552). Required for the maintenance of the epithelial barrier via promoting desmosome-mediated intercellular attachment of suprabasal epithelium to basal cells (By similarity). May play a role in the protein stability of the desmosome plaque components *DSP*, *JUP*, *PKP1*, *PKP2* and *PKP3* (PubMed:22294297). Required for YAP1 localization at the plasma membrane in keratinocytes in response to mechanical strain, via the formation of an interaction complex composed of *DSG3*, *PKP1* and *YWHAG* (PubMed: 31835537). May also be involved in the positive regulation of YAP1 target gene transcription and as a result cell proliferation (PubMed: 31835537). Positively regulates cellular contractility and cell junction formation via organization of cortical F-actin bundles and anchoring of actin to tight junctions, in conjunction with *RAC1* (PubMed:22796473). The cytoplasmic pool of *DSG3* is required for the localization of *CDH1* and CTNNB1 at developing adherens junctions, potentially via modulation of SRC activity (PubMed: 22294297). Inhibits keratinocyte migration via suppression of p38MAPK signaling, may therefore play a role in moderating wound healing

				(PubMed:26763450). Epidermis, tongue, tonsil, esophagus and carcinomas. Expressed in skin and mucosa (at protein level) (PubMed: 22294297, PubMed: 30528827). Expressed in the basal layer of the outer root sheath of the telogen hair club, specifically at the cell membrane between the apex of the cells and the surrounding hair club (at protein level) (PubMed: 9701552). Expression is less abundant between the lateral margins of the outer root sheath basal cells (at protein level) (PubMed:9701552).
Haptoglobin isoform 2	*HPT*	P00738	*HP*	As a result of hemolysis, hemoglobin is found to accumulate in the kidney and is secreted in the urine. Haptoglobin captures, and combines with free plasma hemoglobin to allow hepatic recycling of heme iron and to prevent kidney damage. Haptoglobin also acts as an antioxidant, has antibacterial activity, and plays a role in modulating many aspects of the acute phase response. Hemoglobin/haptoglobin complexes are rapidly cleared by the macrophage CD163 scavenger receptor expressed on the surface of liver Kupfer cells through an endocytic lysosomal degradation pathway. The uncleaved form of allele alpha-2 (2-2), known as zonulin, plays a role in intestinal permeability, allowing intercellular tight junction disassembly, and controlling the equilibrium between tolerance and immunity to non-self antigens. PubMeD:21248165. Expressed by the liver and secreted in plasma.
Retinol-binding protein 4	*RBP4*	P02753	*RBP4*	Retinol-binding protein that mediates retinol transport in blood plasma (PubMed:5541771). Delivers retinol from the liver stores to the peripheral tissues (Probable). Transfers the bound all-trans retinol to STRA6, that then facilitates retinol transport across the cell membrane (PubMed:22665496).
Regenerating islet-derived protein 3-alpha	*REG3A*	Q06141	*PAP1*	Bactericidal C-type lectin which acts exclusively against Gram-positive bacteria and mediates bacterial killing by binding to surface-exposed carbohydrate moieties of peptidoglycan (PubMed: 16931762). Binds membrane phospholipids and kills bacteria by forming a hexameric membrane-permeabilizing oligomeric pore (PubMed: 24256734). Acts as a hormone in response to different stimuli like anti-inflammatory signals, such as IL17A, or gut microbiome. Secreted by different cell types to activate its receptor EXTL3 and induce cell specific signaling pathways (PubMed: 19158046, PubMed: 22727489, PubMed: 27830702, PubMed: 34099862). Induced by IL17A in keratinocytes, regulates keratinocyte proliferation and differentiation after skin injury via activation of EXTL3-PI3K-AKT signaling pathway (PubMed: 22727489). In parallel, inhibits skin inflammation through the inhibition of inflammatory cytokines such as IL6 and TNF (PubMed: 27830702). In pancreas, is able to permealize beta-cells membrane and stimulate their proliferation (PubMed: 19158046). Expressed by keratinocytes (PubMed: 27830702). Highly expressed in epidermal keratinocytes of psoriasis patients (at protein level) (PubMed: 22727489). Constitutively expressed in intestine. Low expression is found in healthy pancreas.

				Overexpressed during the acute phase of pancreatitis and in some patients with chronic pancreatitis (PubMed:1469087).
Programmed cell death 1 ligand 2	*PD-L2*	Q9BQ51	*PDCD1LG2*	Involved in the costimulatory signal, essential for T-cell proliferation and IFNG production in a PDCD1-independent manner. Interaction with PDCD1 inhibits T-cell proliferation by blocking cell cycle progression and cytokine production (By similarity). Highly expressed in heart, placenta, pancreas, lung and liver and weakly expressed in spleen, lymph nodes and thymus.
Defensin-5	*DEFA5*	Q01523	*DEFA5*	Host-defense peptide that maintains sterility in the urogenital system (PubMed: 12021776, PubMed: 12660734, PubMed: 15616305, PubMed: 19589339, PubMed: 22359618, PubMed: 22573326, PubMed: 25354318, PubMed: 25782105, PubMed: 30808760). Has antimicrobial activity against a wide range of bacteria, including Gram-negative *E. coli*, *P. aeruginosa* and *S. typhimurium*, and Gram-positive *E. aerogenes*, *S. aureus*, *B. cereus*, *E. faecium* and *L. monocytogenes* (PubMed: 12021776, PubMed: 15616305, PubMed: 19589339, PubMed: 22359618, PubMed: 22573326, PubMed: 25354318, PubMed: 30808760). Confers resistance to intestinal infection by *S. typhimurium* (PubMed: 12660734). Exhibits antimicrobial activity against enteric commensal bacteria such as *B. adolescentis*, *L. acidophilus*, *B. breve*, *L. fermentum*, *B. longum* and *S. thermophilus* (PubMed: 25354318). Binds to bacterial membranes and causes membrane disintegration (PubMed: 25782105). Induces the secretion of the chemokine IL-8 by intestinal epithelial cells (PubMed: 19589339). Binds to *B. antracis* lef/lethal factor, a major virulence factor from *B. anthracis*, and neutralizes its enzymatic activity (PubMed: 22573326).

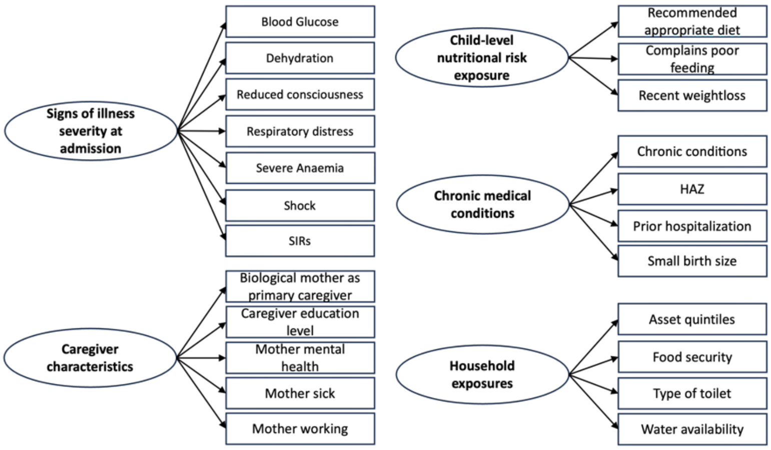

Fig. S 1. *Domains and variables tested for association with 90-days post-discharge weight gain among children after hospitalisation for acute illness in the structural equation model analyses. HAZ = Height-For-Age Z-Score, SIRs = Systemic Inflammatory Response Syndrome.*

Supplementary Results

Table S 3. *Baseline demographic, anthropometric, and clinical characteristics stratified by inclusion status*

Variable	Inclusion status: Included (n=550)	Inclusion status: Excluded (n=96)	p-value
**Demographic**			
Age (months)	11.3 (7.1 to 16.1)	10.8 (6.3 to 15.5)	0.4269
Sex: Female, (%)	223 (41%)	40 (42%)	0.8365
Site n (%)			2.54e-11
Banfora	81 (15%)	6 (6.3%)	
Blantyre	50 (9.1%)	24 (25%)	
Dhaka	88 (16%)	0 (0%)	
Kampala	83 (15%)	15 (16%)	
Karachi	48 (8.7%)	25 (26%)	
Kilifi	46 (8.4%)	5 (5.2%)	
Matlab	61 (11%)	11 (11%)	
Migori	45 (8.2%)	1 (1.0%)	
Nairobi	48 (8.7%)	9 (9.4%)	
**Anthropometric indices**			
WAZ Med. (IQR)	-2.40 (-3.52 to -1.27)	-1.95 (-2.91 to -1.00)	0.0102
MUAC (cm) Med. (IQR)	12.20 (11.35 to 13.25)	12.50 (11.65 to 14.00)	0.0515
WHZ Med. (IQR)	-1.76 (-2.75 to -0.79)	-1.61 (-2.73 to -0.37)	0.2617
HAZ Med. (IQR)	-1.96 (-3.09 to -1.10)	-1.56 (-2.19 to -0.71)	0.0016
WAD at Discharge Med. (IQR)	-2.14 (-3.11 to -1.16)	-1.81 (-2.69 to -0.94)	0.0070
WAZ at 3m post-discharge Med. (IQR)	-1.87 (-2.89 to -1.02)	-1.50 (-2.34 to -0.66)	0.0099
WAD at 3m post-discharge Med. (IQR)	-1.88 (-2.85 to -1.07)	-1.56 (-2.15 to -0.73)	0.0062
Delta-WAZ at 3m post-discharge Med. (IQR)	0.37 (-0.02 to 0.92)	0.45 (0.03 - 1.00)	0.5692
Delta-WAD at 3m post-discharge Med. (IQR)	0.17 (-0.18 to 0.61)	0.22 (-0.11 to 0.73)	0.4304
**Length of hopitalization**			
Days in hospital Med. (IQR)	4.0 (3.0 to 7.0)	3.0 (1.0 to 6.0)	1.20e-05
**Clinical and Haematology**			
Albumin, g/L; Med. (IQR)	39.0 (35.6 to 42.0)	38.0 (34.0 to 43.5)	0.8571
Haemoglobin, g/dL; Med. (IQR)	9.60 (8.50 to 10.50)	9.50 (9.00 to 10.30)	0.8916
RBC, $x10^6$/µL; Med. (IQR)	4.40 (3.79 to 4.86)	4.24 (4.02 to 4.59)	0.5320
WBC, $x10^3$/µL; Med. (IQR)	12.3 (9.5 to 15.8)	11.7 (9.2 to 12.9)	0.5833
Platelets, $x10^3$/µL; Med. (IQR)	444 (284 to 590)	301 (204 to 499)	0.0997
Neutrophils, $x10^3$/µL; Med. (IQR)	2.95 (1.98 to 4.43)	4.00 (2.87 to 5.00)	0.2360
Lymphocytes, $x10^3$/µL; Med. (IQR)	7.6 (5.5 to 9.9)	7.0 (5.0 to 7.6)	0.1387
Eosinophils, $x10^3$/µL; Med. (IQR)	0.21 (0.09 to 0.50)	0.12 (0.00 to 0.49)	0.2307
Monocytes, $x10^3$/µL; Med. (IQR)	0.90 (0.56 to 1.22)	0.93 (0.31 to 1.00)	0.3463
Basophils, $x10^3$/µL; Med. (IQR)	0.05 (0.02 to 0.14)	0.00 (0.00 to 0.05)	0.0014
**Biochemistry**			
Alanine transaminase, IU/L; Med. (IQR)	25 (16 to 37)	21 (16 to 26)	0.2102
Alkaline Phosphatase, IU/L; Med. (IQR)	189 (146 to 250)	226 (205 to 276)	0.0947
Blood urea nitrogen, Mmol/L; Med. (IQR)	1.79 (1.18 to 2.50)	1.54 (1.07 to 2.14)	0.4724
Creatinine, µmol/L; Med. (IQR)	19 (16 to 24)	18 (18 to 27)	0.9970
Bilirubin, µmol/µL; Med. (IQR)	3.7 (3.0 to 5.2)	3.4 (3.1 to 4.0)	0.9102
Phosphate, IU/L; Med. (IQR)	1.68 (1.45 to 1.87)	1.52 (1.31 to 1.84)	0.5356
Magnesium, Mmol/L; Med. (IQR)	0.90 (0.83 to 0.99)	0.94 (0.86 to 0.98)	0.2595
Calcium, Mmol/L; Med. (IQR)	2.48 (2.37 to 2.60)	2.71 (2.20 to 3.06)	0.3997

**Clinical illness at admission – *N* (%)**			
Pneumonia	225 (41%)	30 (31%)	0.0740
Diarrhoea	306 (56%)	47 (49%)	0.2252
Sepsis	63 (11%)	20 (21%)	0.0113
Malaria	92 (17%)	15 (16%)	0.7887
Anaemia	108 (20%)	19 (20%)	0.9718
Pulmonary Tuberculosis	8 (1.5%)	1 (1.0%)	1.0000
**Nutritional category at admission – *N* (%)**			
Not wasted	208 (38%)	48 (50%)	
Moderately wasted	145 (26%)	22 (23%)	0.0727
Severely wasted	197 (36%)	26 (27%)	
**Enteric biomarkers of inflammation and permeability**			
Myeloperoxidase ng/ml	1,395.50 (603.00, 3,281.50); 135[3]	1,558.25 (722.00, 4,263.50); 62[3]	0.8582
Calprotectin ug/ml	172.99 (70.42, 369.69); 143[3]	150.41 (66.06, 393.74); 63[3]	0.9693
Alpha-1-Antitrypsin ug/ml	144.08 (69.20, 278.59); 138[3]	154.49 (78.68, 255.05); 62[3]	0.7792
Lipopolysaccharides EU/ml	2.43 (0.35, 5.24); 17[3]	2.52 (1.59, 3.45); 94[3]	0.9725

IQR = Interquartile Range, WAZ = Weight-For-Age Z Score, MUAC= Mid-upper arm circumference, WHZ= weight-for-length/height z-score, HAZ= Height-For-Age Z-Score, WAD = Weight Absolute Deficit, Delta-WAZ = Change in WAZ, Delta-WAD = Change in WAD, RBC = Red Blood Cells, WBC = White Blood Cells. [1] n (%), [2] Wilcoxon rank sum test; Pearson's Chi-squared test; Fisher's exact test; - two sided tests, [3] Missing

Table S 4: Baseline demographic, anthropometric, clinical characteristics and biomarkers stratified by sex

Characteristic	*Female* *N = 223*[1]	*Male* *N = 327*[1]	*p-value*[2]
Age (Discharge)	11.59 (5.52) | 10.90 (7.10, 15.40)	12.12 (5.92) | 11.80 (7.10, 16.50)	0.3665
**Nutritional status**			
Not Wasted No. (%)	80 (36)	128 (39)	
Moderately Wasted No. (%)	53 (24)	92 (28)	0.1744
Severely Wasted No. (%)	90 (40)	107 (33)	
**Anthropometry**			
WAZ	-2.25 (1.58) | -2.21 (-3.43, -1.11)	-2.55 (1.63) | -2.55 (-3.69, -1.44)	0.0262
MUAC (cm)	12.10 (1.52) | 12.05 (11.15, 13.15)	12.30 (1.54) | 12.25 (11.45, 13.35)	0.1001
WHZ	-1.61 (1.45) | -1.46 (-2.50, -0.65)	-1.84 (1.54) | -1.90 (-2.89, -0.98)	0.0183
HAZ	-1.83 (1.47) | -1.83 (-2.82, -0.98)	-2.21 (1.69) | -2.09 (-3.31, -1.15)	0.0080
WAD at Discharge	-1.96 (1.37) | -1.94 (-2.96, -1.01)	-2.29 (1.44) | -2.28 (-3.29, -1.35)	0.0063
WAZ at 3m post-discharge	-1.75 (1.36) | -1.74 (-2.65, -0.85) | 23[3]	-2.11 (1.44) | -2.01 (-3.03, -1.16) | 37[3]	0.0081
WAD at 3m post-discharge	-1.72 (1.32) | -1.75 (-2.48, -0.90) | 23[3]	-2.09 (1.38) | -2.03 (-3.06, -1.23) | 37[3]	0.0036
Delta-WAZ at 3m post-discharge	0.49 (0.82) | 0.36 (-0.01, 0.92) | 23[3]	0.47 (0.77) | 0.38 (-0.02, 0.92) | 37[3]	0.9627
Delta-WAD at 3m post-discharge	0.26 (0.67) | 0.15 (-0.15, 0.65) | 23[3]	0.23 (0.68) | 0.18 (-0.19, 0.58) | 37[3]	0.7206
**Haematology**			
Albumin, g/ L;	38.58 (7.30) | 39.60 (35.87, 42.71) | 20[3]	38.19 (5.46) | 38.64 (35.20, 41.58) | 25[3]	0.1650
Haemoglobin, g/dL;	9.58 (1.53) | 9.85 (8.80, 10.40) | 19[3]	9.40 (1.84) | 9.50 (8.40, 10.55) | 23[3]	0.2230
RBC, $x10^6$/µL;	4.24 (0.81) | 4.30 (3.73, 4.80) | 19[3]	4.30 (0.95) | 4.44 (3.84, 4.91) | 23[3]	0.1700
WBC, $x10^3$/µL;	12.93 (4.72) | 12.30 (9.60, 15.64) | 19[3]	13.34 (5.84) | 12.27 (9.32, 16.10) | 23[3]	0.9253

Platelets, x10^{3}/μL;	463.12 (227.37) | 443.50 (284.00, 602.50) | 19[3]	448.36 (220.13) | 444.00 (284.00, 569.50) | 23[3]	0.5188
Neutrophils, x10^{3}/μL;	3.38 (2.04) | 2.98 (2.00, 4.25) | 34[3]	3.64 (2.69) | 2.95 (1.82, 4.68) | 47[3]	0.9093
Lymphocytes, x10^{3}/μL;	8.05 (3.39) | 7.76 (5.70, 9.73) | 19[3]	8.08 (3.84) | 7.53 (5.50, 9.90) | 23[3]	0.8093
Eosinophils, x10^{3}/μL;	0.31 (0.42) | 0.17 (0.08, 0.39) | 34[3]	0.46 (0.63) | 0.24 (0.10, 0.60) | 47[3]	0.0108
Monocytes, x10^{3}/μL;	0.94 (0.57) | 0.91 (0.55, 1.16) | 34[3]	1.03 (0.69) | 0.89 (0.58, 1.27) | 47[3]	0.3494
Basophils, x10^{3}/μL;	0.11 (0.14) | 0.06 (0.02, 0.15) | 34[3]	0.11 (0.21) | 0.05 (0.02, 0.12) | 47[3]	0.3417
**Systemic inflammation**			
CCL21	2.97 (0.51) | 2.99 (2.67, 3.30)	3.08 (0.57) | 3.12 (2.72, 3.46)	0.0339
CFHR5	3.67 (0.65) | 3.80 (3.40, 4.09)	3.66 (0.68) | 3.79 (3.39, 4.10)	0.9169
IL1RAP	2.93 (0.59) | 2.93 (2.51, 3.28)	2.85 (0.64) | 2.85 (2.44, 3.27)	0.2238
C8G	3.04 (0.58) | 3.13 (2.66, 3.46)	3.07 (0.63) | 3.13 (2.71, 3.50)	0.6337
ATP1B1	2.33 (0.79) | 2.36 (1.86, 2.87)	2.34 (0.75) | 2.37 (1.82, 2.87)	0.9268
TNF	2.19 (0.86) | 1.98 (1.61, 2.53)	2.08 (0.71) | 1.99 (1.59, 2.40)	0.4550
IFNG	2.20 (0.70) | 2.07 (1.74, 2.49)	2.17 (0.73) | 2.03 (1.70, 2.50)	0.5705
IL1B	1.66 (0.67) | 1.55 (1.14, 2.08)	1.68 (0.65) | 1.62 (1.19, 2.12)	0.5549
IL10	1.79 (0.60) | 1.74 (1.36, 2.23)	1.74 (0.60) | 1.67 (1.30, 2.13)	0.3515
sCD14	2.50 (0.49) | 2.53 (2.26, 2.83)	2.49 (0.53) | 2.55 (2.17, 2.83)	0.9423
LBP	2.73 (0.58) | 2.77 (2.36, 3.11)	2.80 (0.57) | 2.85 (2.46, 3.17)	0.0903
PLA2G2A	1.52 (0.68) | 1.39 (1.07, 1.83)	1.49 (0.73) | 1.31 (1.00, 1.85)	0.2905
CRP	2.82 (0.90) | 2.87 (2.25, 3.60)	3.00 (0.83) | 3.22 (2.50, 3.65)	0.0264
**Growth mediators**			
GHR	2.10 (0.66) | 2.00 (1.68, 2.39)	2.10 (0.60) | 2.04 (1.65, 2.49)	0.6203
THBS4	3.06 (0.63) | 3.07 (2.64, 3.47)	3.03 (0.62) | 3.03 (2.63, 3.48)	0.6641
ACAN	2.24 (0.62) | 2.29 (1.81, 2.65)	2.23 (0.61) | 2.26 (1.80, 2.68)	0.6409
IGFBP6	3.05 (0.61) | 3.09 (2.67, 3.46)	3.01 (0.55) | 3.01 (2.63, 3.38)	0.2970
IGFBP3	3.74 (0.62) | 3.81 (3.35, 4.21)	3.61 (0.64) | 3.73 (3.22, 4.08)	0.0120
IGF2	3.72 (0.58) | 3.82 (3.41, 4.09)	3.59 (0.62) | 3.72 (3.17, 4.06)	0.0241
IGF1	3.23 (0.72) | 3.26 (2.76, 3.76)	3.03 (0.73) | 3.11 (2.56, 3.58)	0.0015
GDF11	2.23 (0.79) | 2.09 (1.67, 2.62)	2.20 (0.80) | 2.00 (1.63, 2.56)	0.3659
CREG1	1.72 (0.67) | 1.65 (1.27, 2.04)	1.87 (0.68) | 1.80 (1.37, 2.26)	0.0081
GDF15	2.82 (0.61) | 2.79 (2.45, 3.20)	2.88 (0.57) | 2.86 (2.51, 3.23)	0.2627
PYY	2.38 (0.60) | 2.33 (1.92, 2.77)	2.40 (0.60) | 2.35 (1.96, 2.84)	0.5390
GCG	2.62 (0.62) | 2.60 (2.20, 3.09)	2.63 (0.59) | 2.63 (2.21, 3.11)	0.9802
IGFBP2	1.84 (0.69) | 1.81 (1.29, 2.31)	1.99 (0.68) | 2.01 (1.46, 2.47)	0.0141
**Enteric inflammation and permeability biomarkers**			
Myeloperoxidase ng/ml	2,764.06 (3,738.35) | 1,360.50 (625.50, 3,526.00) | 54[3]	2,414.71 (2,630.87) | 1,419.00 (579.00, 3,154.00) | 81[3]	0.8986
Calprotectin L ug/ml	320.76 (469.86) | 157.39 (73.07, 293.97) | 57[3]	408.74 (959.64) | 176.03 (70.42, 404.18) | 86[3]	0.5174
Alpha-1-Antitrypsin ug/ml	247.00 (368.65) | 141.64 (64.05, 257.21) | 55[3]	257.60 (335.02) | 144.90 (74.31, 301.18) | 83[3]	0.5014
Lipopolysaccharides EU/ml	4.48 (6.21) | 2.39 (0.32, 5.27) | 6[3]	4.00 (5.40) | 2.49 (0.35, 5.18) | 11[3]	0.9768
FABP2	1.71 (0.92) | 1.51 (1.04, 2.16)	1.56 (0.79) | 1.34 (1.03, 2.00)	0.0620
ZO1	2.01 (0.71) | 1.89 (1.58, 2.35)	2.09 (0.67) | 2.04 (1.63, 2.48)	0.0300
OCLN	2.47 (0.63) | 2.42 (2.03, 2.79)	2.42 (0.61) | 2.37 (1.99, 2.78)	0.4272
CLD1	2.17 (0.62) | 2.15 (1.82, 2.47)	2.21 (0.67) | 2.17 (1.77, 2.59)	0.7061
CDH1	2.67 (0.52) | 2.70 (2.31, 3.06)	2.58 (0.50) | 2.63 (2.27, 2.90)	0.0405
JAMA	1.77 (0.81) | 1.54 (1.20, 2.16)	1.93 (0.87) | 1.68 (1.36, 2.25)	0.0069

DAO	1.74 (1.09) | 1.41 (1.01, 2.01)	1.78 (1.12) | 1.40 (1.03, 2.13)	0.8393
DSG3	1.70 (0.78) | 1.54 (1.14, 2.05)	1.67 (0.71) | 1.55 (1.21, 2.06)	0.9462
HPT	3.49 (1.68) | 4.30 (3.68, 4.51)	3.61 (1.62) | 4.33 (3.81, 4.52)	0.2734
DEFA5	2.42 (0.52) | 2.39 (2.10, 2.78)	2.34 (0.52) | 2.33 (2.00, 2.63)	0.0727
REG3A	2.31 (0.83) | 2.24 (1.82, 2.73)	2.21 (0.85) | 2.15 (1.57, 2.71)	0.0755
PD_L2	2.62 (0.58) | 2.60 (2.26, 3.00)	2.66 (0.54) | 2.66 (2.30, 3.00)	0.3284
RBP4	3.20 (0.51) | 3.25 (2.89, 3.56)	3.11 (0.46) | 3.12 (2.86, 3.39)	0.0064

[1] Mean (SD) | Median (Q1, Q3) [2] Kruskal-Wallis rank sum test [3] Missing data. WAZ = Weight-for-Age Z-score, MUAC= Mid-Upper Arm Circumference, WHZ= Weight-for-Length/Height Z-score, HAZ= Height-for-Age Z-Score, WAD = Weight Absolute Deficit, Delta-WAZ = Change in WAZ, Delta-WAD = Change in WAD, RBC = Red Blood Cells, and WBC = White Blood Cells.

Table S 5: Baseline demographic, anthropometric, clinical characteristics and biomarkers stratified by wasting status

**Characteristic**	**NW** N = 208[1]	**MW** N = 145[1]	**SW** N = 197[1]	**p-value[2]**
Age (Discharge)	12.08 (5.74) | 11.60 (7.15, 16.10)	11.82 (5.37) | 11.90 (7.30, 16.10)	11.80 (6.08) | 10.70 (6.60, 16.40)	0.8283
**Anthropometry**				
WAZ	-1.10 (1.11) | -1.10 (-1.76, -0.49)	-2.48 (0.97) | -2.52 (-3.24, -1.75)	-3.79 (1.25) | -3.77 (-4.68, -3.17)	7.748e-66
MUAC (cm)	13.65 (0.98) | 13.50 (13.03, 14.08)	12.02 (0.43) | 12.05 (11.70, 12.35)	10.85 (1.16) | 10.85 (10.15, 11.45)	4.438e-85
WHZ	-0.61 (1.14) | -0.70 (-1.42, 0.15)	-2.01 (0.99) | -2.18 (-2.73, -1.37)	-2.76 (1.35) | -2.80 (-3.69, -1.90)	4.928e-50
HAZ	-1.14 (1.28) | -1.22 (-1.86, -0.31)	-1.89 (1.23) | -1.86 (-2.63, -1.18)	-3.15 (1.54) | -3.16 (-3.92, -2.20)	1.212e-39
WAD at Discharge	1.05 (1.10) | 1.10 (0.45, 1.78)	2.27 (0.94) | 2.16 (1.59, 3.03)	3.24 (1.10) | 3.17 (2.59, 3.86)	4.865e-58
WAZ at 3m post-discharge	-0.94 (1.02) | -1.02 (-1.53, -0.39) | 25[3]	-2.18 (0.99) | -2.13 (-2.81, -1.50) | 13[3]	-2.87 (1.35) | -2.89 (-3.73, -2.03) | 22[3]	2.217e-40
WAD at 3m post-discharge	-0.98 (1.10) | -1.10 (-1.64, -0.45) | 25[3]	-2.20 (1.01) | -2.10 (-2.98, -1.49) | 13[3]	-2.75 (1.24) | -2.59 (-3.49, -1.95) | 22[3]	2.958e-37
Delta-WAZ at 3m post-discharge	0.17 (0.55) | 0.14 (-0.13, 0.44) | 25[3]	0.32 (0.67) | 0.33 (-0.07, 0.69) | 13[3]	0.91 (0.90) | 0.91 (0.30, 1.49) | 22[3]	1.174e-18
Delta-WAD at 3m post-discharge	0.08 (0.53) | 0.06 (-0.20, 0.38) | 25[3]	0.10 (0.55) | 0.10 (-0.21, 0.42) | 13[3]	0.52 (0.80) | 0.48 (-0.02, 1.00) | 22[3]	2.807e-09
**Haematology**				
Albumin, g/ L;	40.40 (5.45) | 40.30 (37.50, 43.00) | 23[3]	38.78 (4.44) | 39.00 (36.00, 41.55) | 7[3]	35.93 (7.33) | 37.00 (31.20, 41.20) | 15[3]	3.682e-09
Haemoglobin, g/dL;	9.61 (1.67) | 9.70 (8.75, 10.70) | 20[3]	9.51 (1.74) | 9.60 (8.50, 10.50) | 8[3]	9.31 (1.77) | 9.60 (8.20, 10.40) | 14[3]	0.2698
RBC, $x10^6$/μL;	4.47 (0.79) | 4.58 (4.05, 4.95) | 20[3]	4.40 (0.95) | 4.46 (3.96, 4.96) | 8[3]	3.99 (0.89) | 4.00 (3.38, 4.63) | 14[3]	2.903e-08
WBC, $x10^3$/μL;	12.24 (4.90) | 11.33 (8.80, 14.45) | 20[3]	13.83 (5.77) | 12.90 (9.92, 16.88) | 8[3]	13.66 (5.55) | 12.40 (10.05, 16.60) | 14[3]	0.0046
Platelets, $x10^3$/μL;	414.66 (202.22) | 389.00 (265.50, 522.00) | 20[3]	462.14 (233.26) | 460.00 (285.00, 622.00) | 8[3]	489.11 (230.05) | 484.00 (331.00, 628.00) | 14[3]	0.0029
Neutrophils, $x10^3$/μL;	3.12 (2.61) | 2.48 (1.62, 3.79) | 38[3]	3.77 (2.37) | 3.05 (2.08, 5.00) | 15[3]	3.78 (2.29) | 3.30 (2.07, 4.72) | 28[3]	0.0002
Lymphocytes, $x10^3$/μL;	7.51 (3.18) | 7.34 (5.33, 8.97) | 20[3]	8.47 (4.20) | 7.92 (5.60, 10.54) | 8[3]	8.35 (3.64) | 7.76 (5.96, 10.00) | 14[3]	0.0667
Eosinophils, $x10^3$/μL;	0.38 (0.49) | 0.23 (0.09, 0.50) | 38[3]	0.50 (0.66) | 0.26 (0.09, 0.66) | 15[3]	0.35 (0.53) | 0.17 (0.09, 0.38) | 28[3]	0.1480
Monocytes, $x10^3$/μL;	0.92 (0.60) | 0.80 (0.50, 1.16) | 38[3]	0.97 (0.56) | 0.92 (0.62, 1.16) | 15[3]	1.10 (0.74) | 1.00 (0.63, 1.28) | 28[3]	0.0280
Basophils, $x10^3$/μL;	0.10 (0.12) | 0.05 (0.02, 0.12) | 38[3]	0.09 (0.12) | 0.05 (0.02, 0.12) | 15[3]	0.14 (0.26) | 0.06 (0.03, 0.17) | 28[3]	0.1010
**Systemic inflammation**				
CCL21	3.05 (0.59) | 3.09 (2.71, 3.47)	3.09 (0.53) | 3.12 (2.82, 3.46)	2.98 (0.52) | 2.96 (2.62, 3.29)	0.0309
CFHR5	3.91 (0.45) | 3.99 (3.65, 4.21)	3.77 (0.48) | 3.80 (3.54, 4.08)	3.33 (0.82) | 3.50 (2.81, 3.94)	3.878e-15
IL1RAP	3.09 (0.52) | 3.08 (2.74, 3.41)	2.97 (0.63) | 2.97 (2.63, 3.34)	2.60 (0.60) | 2.54 (2.20, 3.00)	6.002e-17
C8G	3.20 (0.56) | 3.28 (2.85, 3.60)	3.10 (0.62) | 3.18 (2.76, 3.47)	2.87 (0.61) | 2.98 (2.50, 3.28)	1.039e-07
ATP1B1	2.45 (0.75) | 2.54 (1.90, 2.98)	2.36 (0.76) | 2.39 (1.86, 2.87)	2.19 (0.76) | 2.22 (1.74, 2.73)	0.0035
TNF	2.21 (0.79) | 2.08 (1.69, 2.52)	2.10 (0.76) | 1.94 (1.57, 2.36)	2.05 (0.76) | 1.88 (1.54, 2.46)	0.0519
IFNG	2.31 (0.71) | 2.17 (1.86, 2.61)	2.05 (0.63) | 1.97 (1.63, 2.41)	2.15 (0.77) | 1.97 (1.71, 2.42)	0.0016

IL1B	1.69 (0.61) | 1.58 (1.25, 2.12)	1.60 (0.68) | 1.47 (1.07, 2.05)	1.71 (0.69) | 1.64 (1.23, 2.23)	0.2434
IL10	1.72 (0.57) | 1.65 (1.32, 2.08)	1.76 (0.65) | 1.67 (1.32, 2.16)	1.81 (0.59) | 1.76 (1.40, 2.28)	0.3157
sCD14	2.49 (0.54) | 2.55 (2.16, 2.84)	2.46 (0.53) | 2.50 (2.21, 2.80)	2.53 (0.47) | 2.55 (2.24, 2.83)	0.6085
LBP	2.77 (0.63) | 2.82 (2.32, 3.18)	2.74 (0.55) | 2.78 (2.41, 3.09)	2.79 (0.54) | 2.82 (2.49, 3.14)	0.7809
PLA2G2A	1.53 (0.74) | 1.36 (1.02, 1.89)	1.58 (0.66) | 1.42 (1.13, 1.83)	1.42 (0.71) | 1.27 (0.96, 1.69)	0.0296
CRP	3.03 (0.88) | 3.36 (2.42, 3.78)	3.04 (0.78) | 3.24 (2.63, 3.65)	2.72 (0.86) | 2.82 (2.14, 3.50)	2.341e-05
**Growth mediators**				
GHR	2.35 (0.60) | 2.32 (1.91, 2.74)	2.07 (0.55) | 2.01 (1.75, 2.29)	1.84 (0.59) | 1.78 (1.45, 2.14)	9.317e-18
THBS4	3.22 (0.55) | 3.20 (2.87, 3.59)	2.97 (0.56) | 2.91 (2.62, 3.35)	2.92 (0.70) | 2.97 (2.40, 3.43)	2.578e-06
ACAN	2.36 (0.59) | 2.35 (1.98, 2.74)	2.25 (0.57) | 2.31 (1.80, 2.63)	2.10 (0.64) | 2.04 (1.62, 2.54)	5.104e-05
IGFBP6	3.04 (0.55) | 3.07 (2.64, 3.39)	2.94 (0.60) | 2.99 (2.63, 3.30)	3.08 (0.57) | 3.09 (2.67, 3.50)	0.1526
IGFBP3	3.84 (0.57) | 3.92 (3.56, 4.21)	3.62 (0.62) | 3.68 (3.34, 4.06)	3.51 (0.66) | 3.62 (3.04, 4.01)	1.365e-07
IGF2	3.77 (0.56) | 3.89 (3.44, 4.20)	3.62 (0.58) | 3.75 (3.33, 3.99)	3.52 (0.65) | 3.70 (3.06, 3.96)	7.006e-05
IGF1	3.35 (0.67) | 3.44 (2.88, 3.81)	3.07 (0.69) | 3.12 (2.64, 3.58)	2.89 (0.76) | 2.98 (2.37, 3.37)	1.762e-09
GDF11	2.21 (0.81) | 2.06 (1.65, 2.52)	2.28 (0.81) | 2.13 (1.69, 2.71)	2.17 (0.78) | 2.07 (1.60, 2.54)	0.4007
CREG1	1.66 (0.55) | 1.61 (1.24, 2.02)	1.69 (0.55) | 1.66 (1.28, 1.96)	2.06 (0.81) | 1.95 (1.46, 2.54)	4.787e-08
GDF15	2.67 (0.59) | 2.67 (2.37, 2.99)	2.83 (0.56) | 2.82 (2.45, 3.15)	3.07 (0.53) | 3.09 (2.71, 3.36)	2.343e-11
PYY	2.24 (0.61) | 2.16 (1.83, 2.61)	2.40 (0.58) | 2.36 (2.00, 2.78)	2.55 (0.57) | 2.51 (2.16, 2.95)	1.187e-07
GCG	2.43 (0.59) | 2.41 (1.99, 2.85)	2.61 (0.63) | 2.65 (2.16, 3.14)	2.85 (0.53) | 2.86 (2.49, 3.22)	4.247e-11
IGFBP2	1.73 (0.64) | 1.73 (1.25, 2.18)	1.89 (0.69) | 1.97 (1.33, 2.33)	2.16 (0.68) | 2.19 (1.66, 2.71)	9.317e-18
**Enteric inflammation and permeability biomarkers**				
Myeloperoxidase ng/ml	2,431.98 (2,405.11) | 1,574.50 (600.00, 3,527.50) | 65[3]	2,681.64 (3,523.85) | 1,403.25 (609.75, 2,908.25) | 37[3]	2,583.87 (3,415.70) | 1,280.50 (606.25, 3,070.25) | 33[3]	0.7573
Calprotectin L ug/ml	400.84 (1,126.22) | 154.43 (65.82, 345.20) | 67[3]	345.75 (484.17) | 180.62 (91.12, 368.71) | 40[3]	366.03 (591.92) | 172.99 (72.77, 392.57) | 36[3]	0.0559
Alpha-1-Antitrypsin ug/ml	274.21 (320.84) | 171.50 (84.15, 307.93) | 66[3]	257.75 (384.69) | 146.03 (72.95, 299.33) | 38[3]	232.10 (348.20) | 121.73 (56.10, 230.15) | 34[3]	0.5547
Lipopolysaccharides EU/ml	3.30 (5.30) | 2.10 (0.27, 4.05) | 5[3]	4.28 (5.83) | 2.00 (0.27, 6.04) | 6[3]	5.09 (6.01) | 3.50 (0.63, 7.64) | 6[3]	0.0049
FABP2	1.59 (0.82) | 1.35 (1.03, 1.95)	1.48 (0.79) | 1.33 (0.98, 1.86)	1.76 (0.90) | 1.55 (1.13, 2.24)	0.0054
ZO1	2.14 (0.72) | 2.06 (1.69, 2.47)	2.00 (0.63) | 1.96 (1.54, 2.36)	2.02 (0.69) | 1.88 (1.59, 2.37)	0.0749
OCLN	2.46 (0.61) | 2.40 (2.03, 2.78)	2.53 (0.67) | 2.46 (2.05, 2.89)	2.36 (0.57) | 2.33 (1.96, 2.70)	0.0834
CLD1	2.31 (0.70) | 2.23 (1.84, 2.68)	2.12 (0.58) | 2.11 (1.77, 2.43)	2.13 (0.63) | 2.10 (1.75, 2.46)	0.0233
CDH1	2.52 (0.51) | 2.54 (2.14, 2.89)	2.65 (0.50) | 2.68 (2.32, 2.98)	2.71 (0.51) | 2.71 (2.38, 3.05)	0.0015
JAMA	1.77 (0.83) | 1.56 (1.25, 2.01)	1.91 (0.94) | 1.55 (1.26, 2.28)	1.94 (0.78) | 1.73 (1.38, 2.32)	0.0137
DAO	1.55 (0.91) | 1.30 (1.00, 1.78)	1.62 (1.10) | 1.30 (0.98, 1.68)	2.10 (1.22) | 1.70 (1.13, 2.65)	4.856e-07
DSG3	1.67 (0.69) | 1.56 (1.20, 2.08)	1.76 (0.74) | 1.68 (1.21, 2.05)	1.64 (0.80) | 1.44 (1.14, 1.93)	0.1070
HPT	3.83 (1.44) | 4.39 (4.04, 4.57)	3.56 (1.72) | 4.37 (3.97, 4.54)	3.27 (1.74) | 4.14 (3.09, 4.43)	2.336e-06
DEFA5	2.31 (0.47) | 2.32 (2.01, 2.56)	2.43 (0.55) | 2.37 (2.11, 2.79)	2.41 (0.53) | 2.40 (2.02, 2.81)	0.0366
REG3A	2.09 (0.85) | 1.94 (1.50, 2.50)	2.30 (0.87) | 2.21 (1.73, 2.85)	2.38 (0.78) | 2.38 (1.83, 2.84)	0.0001
PD_L2	2.61 (0.53) | 2.63 (2.28, 2.98)	2.60 (0.57) | 2.59 (2.22, 2.97)	2.71 (0.57) | 2.68 (2.30, 3.03)	0.2476

RBP4	3.03 (0.46) | 3.05 (2.78, 3.33)	3.07 (0.51) | 3.10 (2.78, 3.37)	3.32 (0.45) | 3.35 (3.05, 3.61)	7.545e-11

[1] Mean (SD) | Median (Q1, Q3) [2] Kruskal-Wallis rank sum test [3] Missing data. WAZ = Weight-for-Age Z-score, MUAC= Mid-Upper Arm Circumference, WHZ= Weight-for-Length/Height Z-score, HAZ= Height-for-Age Z-Score, WAD = Weight Absolute Deficit, Delta-WAZ = Change in WAZ, Delta-WAD = Change in WAD. NW = Not Wasted, MW = Moderately Wasted, SW = Severely Wasted, RBC = Red Blood Cells, and WBC = White Blood Cells.

Table S 6: Baseline demographic, anthropometric, clinical characteristics and biomarkers stratified by age group

Characteristic	< 6 months N = 106[1]	≥6 months to <12months N = 187[1]	≥ 12 months N = 257[1]	p-value[2]
**Nutritional status**				
Not Wasted No. (%)	38 (36)	72 (39)	98 (38)	0.58
Moderately Wasted No. (%)	23 (22)	52 (28)	70 (27)	
Severely Wasted No. (%)	45 (42)	63 (34)	89 (35)	
**Anthropometry**				
WAZ	-2.44 (1.96) | -2.19 (-4.03, -1.08)	-2.26 (1.51) | -2.28 (-3.39, -1.15)	-2.54 (1.52) | -2.63 (-3.53, -1.45)	0.3
MUAC (cm)	11.54 (1.65) | 11.60 (10.35, 12.50)	12.32 (1.50) | 12.25 (11.35, 13.40)	12.43 (1.43) | 12.35 (11.50, 13.45)	<0.001
WHZ	-1.45 (1.74) | -1.37 (-2.58, -0.28)	-1.57 (1.42) | -1.57 (-2.46, -0.63)	-2.00 (1.43) | -2.01 (-2.97, -1.05)	<0.001
HAZ	-1.83 (2.00) | -1.84 (-3.29, -0.78)	-1.91 (1.43) | -1.70 (-2.77, -1.08)	-2.26 (1.55) | -2.12 (-3.17, -1.33)	0.029
WAD at Discharge	-1.65 (1.33) | -1.61 (-2.53, -0.78)	-1.88 (1.23) | -1.97 (-2.79, -1.10)	-2.57 (1.47) | -2.76 (-3.48, -1.58)	<0.001
WAZ at 3m post-discharge	-1.86 (1.60) | -1.71 (-2.93, -0.89) | 15[3]	-1.86 (1.40) | -1.81 (-2.72, -0.93) | 26[3]	-2.07 (1.34) | -2.00 (-2.95, -1.15) | 19[3]	0.3
WAD at 3m post-discharge	-1.47 (1.27) | -1.46 (-2.23, -0.73) | 15[3]	-1.71 (1.27) | -1.71 (-2.50, -0.98) | 26[3]	-2.27 (1.39) | -2.23 (-3.21, -1.34) | 19[3]	<0.001
Delta-WAZ at 3m post-discharge	0.57 (0.91) | 0.41 (-0.03, 1.17) | 15[3]	0.44 (0.76) | 0.33 (-0.02, 0.93) | 26[3]	0.46 (0.76) | 0.37 (0.00, 0.85) | 19[3]	0.6
Delta-WAD at 3m post-discharge	0.17 (0.63) | 0.12 (-0.19, 0.52) | 15[3]	0.20 (0.62) | 0.13 (-0.21, 0.64) | 26[3]	0.29 (0.72) | 0.22 (-0.14, 0.64) | 19[3]	0.4
**Haematology**				
Albumin, g/ L;	38.23 (4.95) | 38.00 (35.00, 42.00) | 14[3]	39.15 (6.56) | 39.75 (36.00, 42.44) | 9[3]	37.78 (6.44) | 38.83 (34.90, 42.00) | 22[3]	0.2
Haemoglobin, g/dL;	9.75 (1.63) | 9.90 (8.80, 10.50) | 7[3]	9.74 (1.46) | 9.80 (8.90, 10.70) | 14[3]	9.16 (1.89) | 9.30 (8.10, 10.30) | 21[3]	0.002
RBC, x10^6/μL;	4.08 (0.71) | 4.09 (3.64, 4.63) | 7[3]	4.42 (0.69) | 4.53 (4.00, 4.87) | 14[3]	4.25 (1.07) | 4.38 (3.67, 4.96) | 21[3]	0.001
WBC, x10^3/μL;	12.15 (5.13) | 11.30 (8.90, 14.62) | 7[3]	13.62 (5.44) | 12.50 (10.30, 15.87) | 14[3]	13.29 (5.49) | 12.40 (9.07, 16.16) | 21[3]	0.038
Platelets, x10^3/μL;	447.75 (203.79) | 472.00 (286.00, 591.00) | 7[3]	468.56 (221.60) | 438.00 (336.00, 600.00) | 14[3]	446.56 (231.87) | 414.50 (268.50, 575.00) | 21[3]	0.5
Neutrophils, x10^3/μL;	2.92 (1.75) | 2.53 (1.69, 3.75) | 23[3]	3.60 (2.62) | 2.80 (1.95, 4.35) | 25[3]	3.72 (2.51) | 3.25 (2.00, 4.90) | 33[3]	0.034
Lymphocytes, x10^3/μL;	7.41 (4.01) | 6.50 (5.00, 8.71) | 7[3]	8.53 (3.61) | 8.03 (6.46, 10.10) | 14[3]	8.01 (3.52) | 7.36 (5.42, 9.92) | 21[3]	0.006
Eosinophils, x10^3/μL;	0.39 (0.50) | 0.24 (0.09, 0.45) | 23[3]	0.33 (0.44) | 0.20 (0.08, 0.42) | 25[3]	0.45 (0.65) | 0.20 (0.09, 0.57) | 33[3]	0.5
Monocytes, x10^3/μL;	0.85 (0.51) | 0.81 (0.49, 1.13) | 23[3]	0.99 (0.66) | 0.86 (0.53, 1.15) | 25[3]	1.06 (0.67) | 1.00 (0.63, 1.29) | 33[3]	0.036

Basophils, $x10^3$/µL;	0.07 (0.07) | 0.04 (0.02, 0.09) | 23[3]	0.14 (0.26) | 0.05 (0.03, 0.17) | 25[3]	0.11 (0.13) | 0.06 (0.02, 0.14) | 33[3]	0.045
**Systemic inflammation**				
CCL21	3.01 (0.54) | 2.97 (2.58, 3.38)	3.06 (0.60) | 3.09 (2.72, 3.49)	3.03 (0.51) | 3.07 (2.72, 3.36)	0.4
CFHR5	3.43 (0.58) | 3.55 (3.19, 3.80)	3.63 (0.68) | 3.76 (3.41, 4.04)	3.79 (0.66) | 3.97 (3.58, 4.19)	<0.001
IL1RAP	2.76 (0.57) | 2.77 (2.44, 3.09)	2.93 (0.59) | 2.94 (2.53, 3.30)	2.90 (0.66) | 2.91 (2.44, 3.34)	0.037
C8G	2.82 (0.56) | 2.89 (2.42, 3.26)	3.07 (0.60) | 3.14 (2.75, 3.49)	3.14 (0.62) | 3.19 (2.81, 3.56)	<0.001
ATP1B1	2.46 (0.85) | 2.47 (1.90, 3.18)	2.34 (0.76) | 2.45 (1.86, 2.87)	2.28 (0.73) | 2.26 (1.81, 2.80)	0.064
TNF	1.87 (0.70) | 1.69 (1.39, 2.16)	2.17 (0.86) | 2.02 (1.65, 2.36)	2.19 (0.72) | 2.06 (1.70, 2.54)	<0.001
IFNG	2.06 (0.79) | 1.88 (1.54, 2.44)	2.17 (0.68) | 2.06 (1.73, 2.50)	2.24 (0.71) | 2.10 (1.80, 2.50)	0.011
IL1B	1.65 (0.65) | 1.55 (1.18, 2.06)	1.61 (0.65) | 1.49 (1.12, 2.05)	1.73 (0.66) | 1.67 (1.22, 2.16)	0.2
IL10	1.77 (0.55) | 1.77 (1.38, 2.17)	1.67 (0.61) | 1.58 (1.28, 2.01)	1.82 (0.61) | 1.78 (1.37, 2.34)	0.009
sCD14	2.26 (0.55) | 2.34 (1.91, 2.64)	2.48 (0.51) | 2.52 (2.17, 2.85)	2.61 (0.47) | 2.62 (2.34, 2.87)	<0.001
LBP	2.68 (0.58) | 2.71 (2.34, 3.00)	2.76 (0.60) | 2.83 (2.39, 3.16)	2.81 (0.56) | 2.86 (2.52, 3.17)	0.037
PLA2G2A	1.40 (0.66) | 1.23 (0.99, 1.53)	1.45 (0.77) | 1.25 (0.97, 1.72)	1.59 (0.69) | 1.47 (1.11, 2.01)	0.001
CRP	2.77 (0.87) | 2.98 (2.18, 3.51)	2.77 (0.91) | 2.80 (2.08, 3.63)	3.10 (0.79) | 3.37 (2.65, 3.68)	<0.001
**Growth mediators**				
GHR	1.83 (0.64) | 1.76 (1.42, 2.02)	2.09 (0.55) | 2.04 (1.73, 2.37)	2.21 (0.63) | 2.16 (1.77, 2.64)	<0.001
THBS4	3.60 (0.59) | 3.63 (3.24, 3.97)	3.07 (0.54) | 3.06 (2.72, 3.47)	2.80 (0.54) | 2.83 (2.42, 3.18)	<0.001
ACAN	2.57 (0.67) | 2.60 (2.16, 3.07)	2.33 (0.54) | 2.35 (1.96, 2.72)	2.03 (0.56) | 2.05 (1.62, 2.42)	<0.001
IGFBP6	3.16 (0.58) | 3.17 (2.72, 3.57)	3.11 (0.56) | 3.12 (2.75, 3.47)	2.91 (0.56) | 2.92 (2.56, 3.30)	<0.001
IGFBP3	3.46 (0.71) | 3.62 (3.00, 4.01)	3.68 (0.61) | 3.76 (3.34, 4.12)	3.73 (0.60) | 3.81 (3.37, 4.18)	0.008
IGF2	3.50 (0.73) | 3.67 (2.95, 4.02)	3.71 (0.56) | 3.81 (3.39, 4.13)	3.65 (0.58) | 3.80 (3.31, 4.06)	0.081
IGF1	3.07 (0.84) | 3.24 (2.48, 3.68)	3.13 (0.69) | 3.20 (2.68, 3.60)	3.12 (0.71) | 3.14 (2.64, 3.67)	>0.9
GDF11	2.22 (0.71) | 2.14 (1.66, 2.58)	2.36 (0.85) | 2.14 (1.80, 2.72)	2.11 (0.78) | 1.96 (1.58, 2.45)	0.003
CREG1	1.91 (0.64) | 1.86 (1.49, 2.19)	1.74 (0.64) | 1.68 (1.31, 2.07)	1.82 (0.72) | 1.68 (1.33, 2.19)	0.10
GDF15	2.82 (0.61) | 2.85 (2.49, 3.18)	2.88 (0.52) | 2.90 (2.53, 3.21)	2.85 (0.62) | 2.81 (2.44, 3.22)	0.6
PYY	2.47 (0.57) | 2.45 (2.06, 2.82)	2.48 (0.58) | 2.45 (2.05, 2.91)	2.30 (0.62) | 2.23 (1.88, 2.67)	<0.001
GCG	2.56 (0.64) | 2.61 (1.97, 3.02)	2.72 (0.58) | 2.75 (2.31, 3.17)	2.58 (0.60) | 2.54 (2.18, 3.08)	0.032
IGFBP2	1.88 (0.78) | 1.87 (1.23, 2.47)	1.94 (0.62) | 2.02 (1.46, 2.33)	1.94 (0.70) | 1.91 (1.40, 2.44)	0.7
**Enteric inflammation and permeability biomarkers**				
Myeloperoxidase ng/ml	2,992.17 (3,075.72) | 1,999.50 (927.50, 4,239.00) | 31[3]	2,218.66 (2,904.31) | 1,124.75 (517.50, 2,345.00) | 41[3]	2,643.34 (3,298.88) | 1,579.25 (623.00, 3,317.00) | 63[3]	0.019
Calprotectin L ug/ml	407.07 (589.73) | 175.47 (96.10, 493.97) | 31[3]	409.91 (1,165.14) | 147.24 (70.42, 284.71) | 45[3]	331.66 (462.63) | 179.30 (66.26, 383.98) | 67[3]	0.4
Alpha-1-Antitrypsin ug/ml	288.68 (376.06) | 174.63 (92.00, 299.33) | 31[3]	229.23 (343.56) | 131.55 (63.50, 259.40) | 42[3]	257.61 (341.98) | 147.59 (69.08, 291.83) | 65[3]	0.2
Lipopolysaccharides EU/ml	5.97 (8.43) | 2.83 (0.38, 7.37) | 4[3]	3.62 (5.41) | 1.55 (0.02, 4.52) | 5[3]	3.89 (4.34) | 2.69 (0.66, 5.18) | 8[3]	0.020
FABP2	1.65 (1.05) | 1.27 (0.96, 2.04)	1.65 (0.79) | 1.47 (1.08, 2.11)	1.59 (0.80) | 1.40 (1.02, 1.95)	0.3
ZO1	1.89 (0.61) | 1.83 (1.49, 2.21)	2.08 (0.70) | 1.93 (1.60, 2.37)	2.12 (0.70) | 2.06 (1.70, 2.44)	0.005

OCLN	2.51 (0.66) | 2.44 (2.08, 2.83)	2.43 (0.57) | 2.42 (1.99, 2.80)	2.42 (0.63) | 2.37 (1.99, 2.70)	0.4
CLD1	2.13 (0.68) | 2.10 (1.73, 2.45)	2.20 (0.64) | 2.16 (1.77, 2.48)	2.22 (0.65) | 2.21 (1.85, 2.62)	0.2
CDH1	2.48 (0.54) | 2.53 (2.09, 2.80)	2.64 (0.49) | 2.65 (2.31, 2.96)	2.66 (0.51) | 2.68 (2.35, 3.02)	0.011
JAMA	1.75 (0.84) | 1.56 (1.26, 1.96)	1.93 (0.82) | 1.72 (1.36, 2.21)	1.87 (0.86) | 1.61 (1.25, 2.25)	0.064
DAO	1.91 (1.09) | 1.58 (1.21, 2.31)	1.82 (1.14) | 1.47 (1.02, 2.19)	1.66 (1.08) | 1.32 (1.00, 1.83)	0.012
DSG3	1.62 (0.71) | 1.48 (1.11, 1.97)	1.76 (0.77) | 1.67 (1.22, 2.13)	1.65 (0.73) | 1.55 (1.17, 1.95)	0.2
HPT	3.42 (1.62) | 4.16 (3.43, 4.45)	3.90 (1.40) | 4.42 (4.13, 4.56)	3.37 (1.77) | 4.31 (3.37, 4.50)	<0.001
DEFA5	2.37 (0.57) | 2.40 (1.92, 2.74)	2.38 (0.51) | 2.35 (2.09, 2.68)	2.38 (0.51) | 2.37 (2.05, 2.67)	>0.9
REG3A	1.89 (0.82) | 1.85 (1.27, 2.48)	2.23 (0.85) | 2.14 (1.59, 2.68)	2.41 (0.80) | 2.36 (1.83, 2.85)	<0.001
PD_L2	2.42 (0.49) | 2.42 (2.10, 2.83)	2.60 (0.58) | 2.59 (2.21, 3.00)	2.77 (0.53) | 2.73 (2.41, 3.07)	<0.001
RBP4	3.07 (0.51) | 3.13 (2.79, 3.39)	3.18 (0.52) | 3.21 (2.89, 3.48)	3.15 (0.44) | 3.14 (2.86, 3.43)	0.2

[1] Mean (SD) | Median (Q1, Q3) [2] Kruskal-Wallis rank sum test [3] Missing data. WAZ = Weight-for-Age Z-score, MUAC= Mid-Upper Arm Circumference, WHZ= Weight-for-Length/Height Z-score, HAZ= Height-for-Age Z-Score, WAD = Weight Absolute Deficit, Delta-WAZ = Change in WAZ, Delta-WAD = Change in WAD.

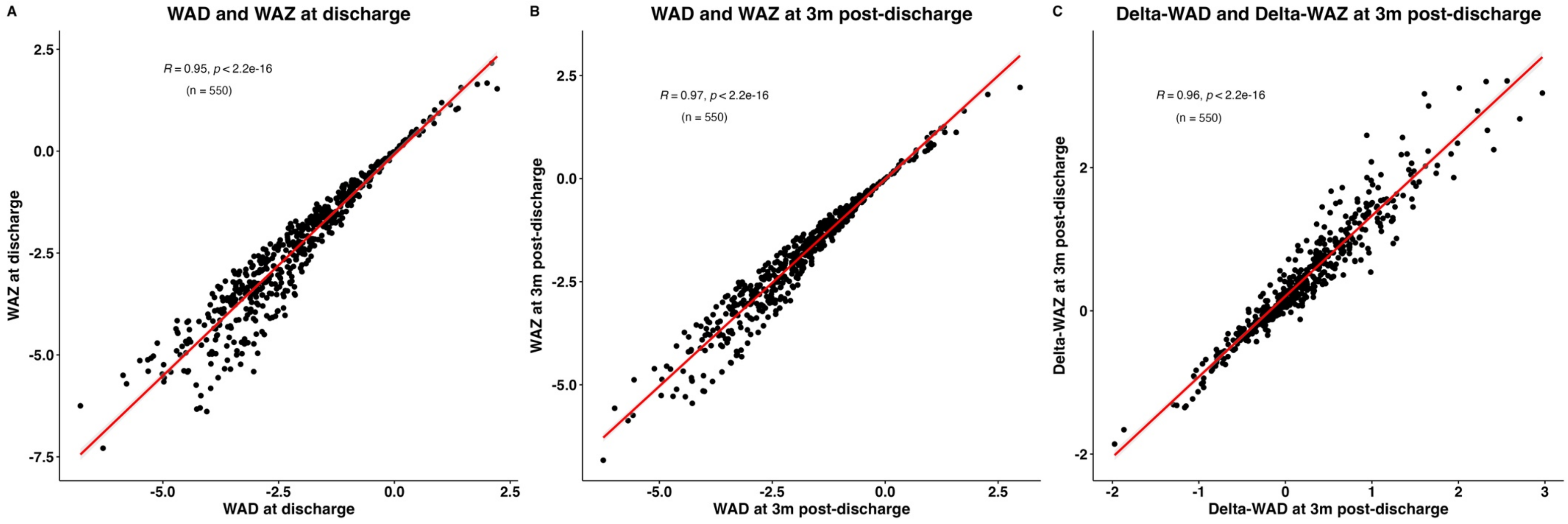

Figure S 2: Scatter plots showing the relationships between WAD and WAZ at discharge (A) and at 3 months post-discharge (B) and between Delta WAD and Delta WAZ at 3 months post-discharge (C) among study children. WAZ = Weight-for-Age Z-score, WAD = Weight Absolute Deficit, Delta-WAZ = Change in WAZ, Delta-WAD = Change in WAD.

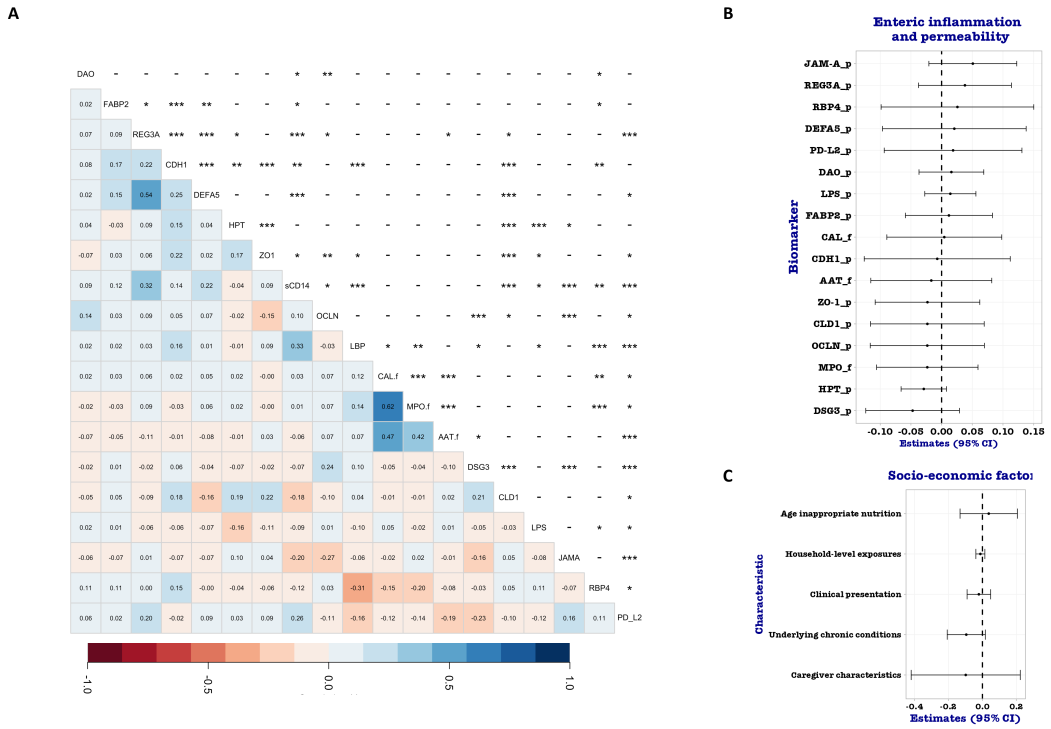

Figure S 3: ***Biomarkers for enteric inflammation and permeability and factors assessed for socio-economic and chronic medical conditions****. A. Correlation plot among the biomarkers for enteric inflammation and permeability. The significance level for correlations are coded as "***" for p<0.0005, "**" for p<0.005, "*" for p<0.05 and "-" for p≥0.05. The variables are ordered according to the PCA-based re-ordering in the corrgram package in R. Biomarkers measured in stool are coded with a suffix ".f" for faecal. Forest plots showing results from generalised linear models testing the association between the biomarkers for enteric inflammation and permeability (B) and socio-economic and chronic medical conditions (C) and weight gain after adjusting for WAD at discharge, sex, age, receipt of therapeutic feeds and site. In panel B, biomarkers measured in plasma are denoted by the suffix "_p" while those measured in stool are denoted by the suffix "_f". FABP2 = Intestinal fatty acid-binding protein, ZO-1 = Tight junction protein ZO-1, OCLN = Occludin, CLD1 = Claudin-1, CDH1 = Cadherin E, JAMA = Junctional adhesion molecule A, DSG3 = Desmoglein-3, REG3A = Regenerating islet-derived protein 3-alpha, DEFA5 = Defensin-5, DAO = D-amino-acid oxidase, HP = Haptoglobin isoform 2, RBP4 = Retinol-binding protein 4, PD-L2 = Programmed cell death 1 ligand 2, MPO = Myeloperoxidase, CAL = Calprotectin, AAT = Alpha-1-Antitrypsin, LPS = Lipopolysaccharides, sCD14 = soluble Monocyte differentiation antigen CD14, LBP = Lipopolysaccharide-binding protein.*

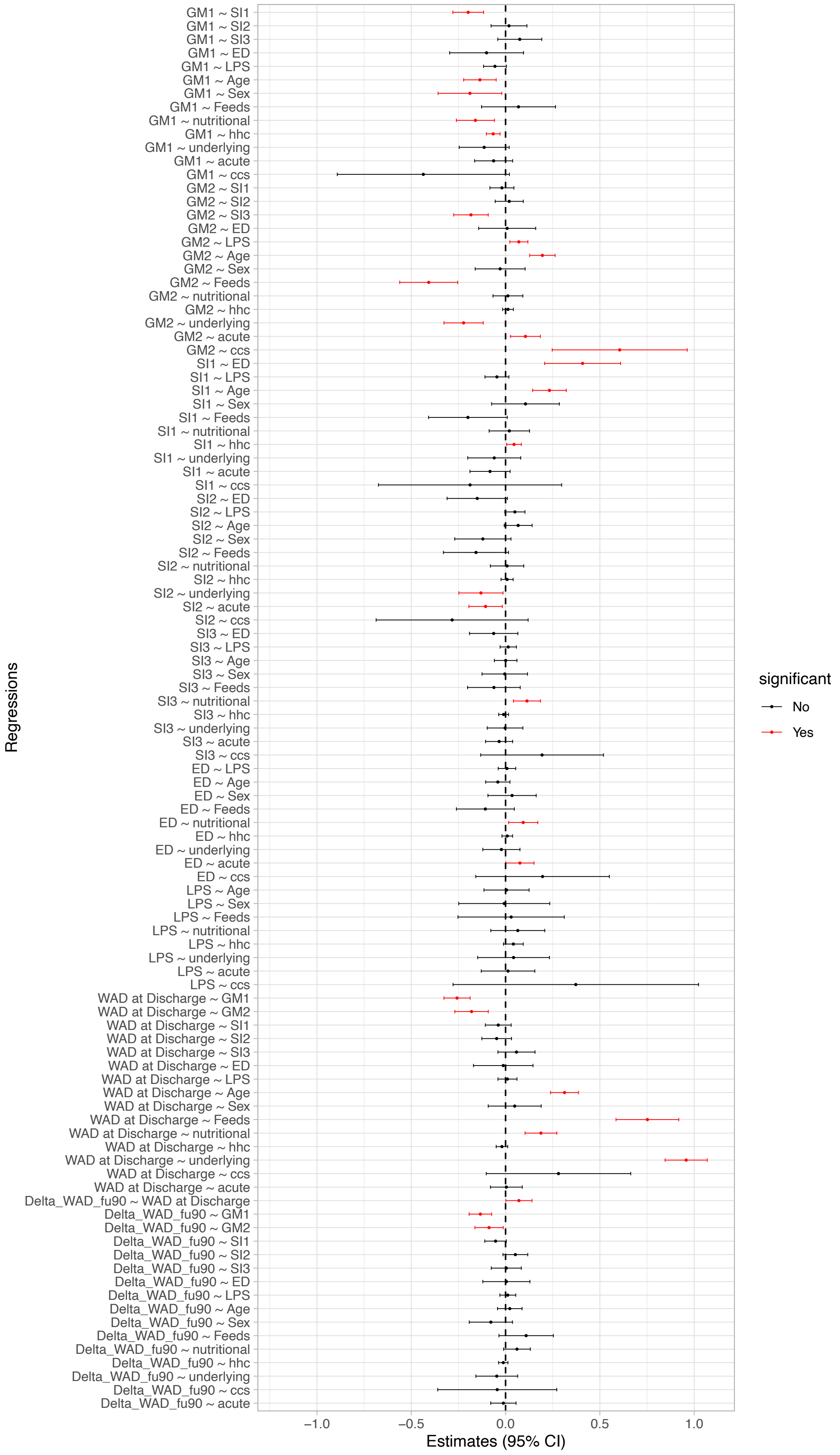
Regressions
Estimates (95% CI)
significant
No
Yes

Figure S 4 ***Biomarkers and relationships with growth using structural equation models.*** *A forest plot showing results from regression analysis from a structural equation model (SEM) examining the relationships between the first three components of both systemic inflammation and growth mediators and growth and how they relate to basal WAD at discharge, receipt of therapeutic and socio-economic, demographic and medical factors. Significant associations ($p<0.05$) in the forest plot are shown in red error bars. SI = systemic inflammation, GM = growth mediators, ED = Enteric Dysfunction, LPS = Lipopolysaccharides, Feeds = receipt of therapeutic feeds, nutritional = age-inappropriate nutrition, hhc = household-level exposures, underlying = underlying chronic conditions, acute = clinical presentation, ccs = caregiver characteristics, and Delta_WAD_fu90 = Delta-WAD at 3m post-discharge.*

Table S 7: Detailed results for the regression analysis from the structural equation model

	Regression	estimate	se	z	pvalue	Lower confidence interval to the Upper confidence interval		fitindices	fitMeasures (sem_waz_fu90)
1	ED=~CAL	1	0	NA	NA	1 to 1		npar	129
2	ED=~MPO	1.00205111	0.08911348	11.2446638	0	0.8273919 to 1.17671032		fmin	0.05130147
3	ED=~AAT	0.66169132	0.06758007	9.79121931	0	0.52923681 to 0.79414583		chisq	56.4316188
4	GM1~SI1	-0.1981388	0.04149646	-4.774836	1.80E-06	-0.2794704 to -0.1168072		df	36
5	GM1~SI2	0.01807789	0.04847488	0.3729332	0.70919816	-0.0769311 to 0.11308691		pvalue	0.01630457
6	GM1~SI3	0.07507174	0.05958717	1.25986404	0.20771841	-0.041717 to 0.19186046		baseline.chisq	1460.36766
7	GM1~ED	-0.1008028	0.0999044	-1.0089923	0.31297834	-0.2966118 to 0.09500626		baseline.df	143
8	GM1~LPS	-0.0562712	0.03084478	-1.8243355	0.06810136	-0.1167259 to 0.00418343		baseline.pvalue	0
9	GM1~Age	-0.1360989	0.0439517	-3.0965566	0.00195782	-0.2222427 to -0.0499552		cfi	0.98449057
10	GM1~Sex	-0.1889744	0.08615808	-2.1933454	0.0282825	-0.3578412 to -0.0201077		tli	0.93839311
11	GM1~Feeds	0.06851471	0.10006119	0.68472805	0.49351554	-0.1276016 to 0.26463104		cfi.robust	0.98142899
12	GM1~nutritional	-0.1599146	0.05152421	-3.1036787	0.00191131	-0.2609002 to -0.058929		tli.robust	0.92623183
13	GM1~hhc	-0.0656268	0.01859265	-3.529715	0.00041601	-0.1020677 to -0.0291858		nnfi	0.93839311
14	GM1~underlying	-0.1134017	0.06767877	-1.6755876	0.09381901	-0.2460497 to 0.01924625		rfi	0.84650514
15	GM1~acute	-0.0633905	0.05147019	-1.231596	0.21810004	-0.1642702 to 0.03748924		nfi	0.96135794
16	GM1~ccs	-0.4360841	0.23281454	-1.8730966	0.06105505	-0.8923922 to 0.02022399		pnfi	0.24202018
17	GM2~SI1	-0.019431	0.03249258	-0.5980139	0.54983068	-0.0831153 to 0.04425327		ifi	0.98565566
18	GM2~SI2	0.01939924	0.03804665	0.50988037	0.61013527	-0.0551708 to 0.0939693		rni	0.98449057
19	GM2~SI3	-0.1836107	0.046769	-3.9259065	8.64E-05	-0.2752763 to -0.0919452		nnfi.robust	0.92623183
20	GM2~ED	0.00840484	0.07714194	0.10895298	0.91323979	-0.1427906 to 0.15960026		rni.robust	0.98142899
21	GM2~LPS	0.07025287	0.02443022	2.87565439	0.00403191	0.02237052 to 0.11813523		logl	-6711.9325
22	GM2~Age	0.19509906	0.03444512	5.66405519	1.48E-08	0.12758786 to 0.26261025		unrestricted.logl	NA
23	GM2~Sex	-0.0287836	0.06760073	-0.4257884	0.67026203	-0.1612786 to 0.10371139		aic	13681.8649
24	GM2~Feeds	-0.4075607	0.07849903	-5.1919202	2.08E-07	-0.561416 to -0.2537054		bic	14237.8444
25	GM2~nutritional	0.01228396	0.04041554	0.30394152	0.76117244	-0.066929 to 0.09149696		ntotal	550
26	GM2~hhc	0.01275953	0.01458997	0.87454126	0.38182356	-0.0158363 to 0.04135534		bic2	13828.3437
27	GM2~underlying	-0.2226321	0.0531079	-4.1920724	2.76E-05	-0.3267217 to -0.1185426		rmsea	0.0321232
28	GM2~acute	0.10526879	0.04036988	2.60760724	0.00911775	0.02614528 to 0.18439231		rmsea.ci.lower	0.01403444
29	GM2~ccs	0.60503891	0.18264521	3.31264598	0.00092418	0.24706088 to 0.96301693		rmsea.ci.upper	0.04761318
30	SI1~ED	0.40817835	0.10268183	3.9751762	7.03E-05	0.20692567 to 0.60943103		rmsea.ci.level	0.9
31	SI1~LPS	-0.0459601	0.03242954	-1.4172282	0.15641621	-0.1095208 to 0.01760067		rmsea.pvalue	0.97286823
32	SI1~Age	0.23245556	0.04553606	5.10486723	3.31E-07	0.14320651 to 0.3217046		rmsea.close.h0	0.05
33	SI1~Sex	0.10555667	0.09158149	1.15259829	0.24907531	-0.0739398 to 0.2850531		rmsea.notclose.pvalue	8.41E-09
34	SI1~Feeds	-0.1997438	0.10640456	-1.8772111	0.06048917	-0.4082929 to 0.00880529		rmsea.notclose.h0	0.08
35	SI1~nutritional	0.01984729	0.05464267	0.36321958	0.71644086	-0.0872504 to 0.12694495		rmsea.robust	0.03705552
36	SI1~hhc	0.04488294	0.01976307	2.27105113	0.02314388	0.00614804 to 0.08361784		rmsea.ci.lower.robust	0.01608648
37	SI1~underlying	-0.0601448	0.07171941	-0.8386129	0.40168657	-0.2007123 to 0.08042264		rmsea.ci.upper.robust	0.05497661
38	SI1~acute	-0.0824533	0.05436908	-1.5165479	0.12938088	-0.1890148 to 0.02410813		rmsea.pvalue.robust	0.87479548

39	SI1~ccs	-0.1882362	0.24775737	-0.7597603	0.44739786	-0.6738317 to 0.2973593		rmsea.notclose.pvalue.robust	9.47E-06
40	SI2~ED	-0.1502306	0.0816294	-1.8403981	0.06570982	-0.3102213 to 0.0097601		rmr	0.01497004
41	SI2~LPS	0.049283	0.02727171	1.80711025	0.07074509	-0.0041686 to 0.10273457		rmr_nomean	0.01565483
42	SI2~Age	0.06662939	0.03775816	1.76463576	0.077625	-0.0073752 to 0.14063402		srmr	0.02266194
43	SI2~Sex	-0.1206059	0.07607721	-1.5853095	0.11289603	-0.2697145 to 0.02850266		srmr_bentler	0.02266194
44	SI2~Feeds	-0.1569924	0.08809097	-1.7821624	0.07472275	-0.3296475 to 0.01566271		srmr_bentler_nomean	0.02370413
45	SI2~nutritional	0.00788022	0.04516551	0.17447429	0.86149274	-0.0806425 to 0.09640298		crmr	0.02375906
46	SI2~hhc	0.00797673	0.01639239	0.48661177	0.62653348	-0.0241518 to 0.04010522		crmr_nomean	0.02497686
47	SI2~underlying	-0.1306822	0.05964018	-2.1911775	0.02843895	-0.2475748 to -0.0137896		srmr_mplus	0.02267704
48	SI2~acute	-0.1060104	0.04517698	-2.3465588	0.01894768	-0.1945557 to -0.0174652		srmr_mplus_nomean	0.02369968
49	SI2~ccs	-0.2833063	0.20559603	-1.3779757	0.1682108	-0.6862671 to 0.11965449		cn_05	498.046757
50	SI3~ED	-0.0631185	0.0654109	-0.9649537	0.33456801	-0.1913215 to 0.06508452		cn_01	572.320984
51	SI3~LPS	0.01414463	0.02202712	0.64214616	0.52077829	-0.0290277 to 0.057317		gfi	0.99672266
52	SI3~Age	0.00045734	0.03054657	0.01497195	0.98805456	-0.0594128 to 0.06032753		agfi	0.98097322
53	SI3~Sex	-0.0043422	0.06155549	-0.0705417	0.94376254	-0.1249888 to 0.11630432		pgfi	0.17168429
54	SI3~Feeds	-0.0619888	0.07120765	-0.8705355	0.38400784	-0.2015532 to 0.07757564		mfi	0.98159724
55	SI3~nutritional	0.11329527	0.03654976	3.09975437	0.00193681	0.04165906 to 0.18493148		ecvi	0.57169385
56	SI3~hhc	-0.0104182	0.01325652	-0.7858948	0.4319291	-0.0364005 to 0.01556407			
57	SI3~underlying	-0.0027486	0.04826271	-0.0569514	0.95458388	-0.0973418 to 0.09184454			
58	SI3~acute	-0.0342125	0.03655455	-0.9359305	0.34930895	-0.1058581 to 0.03743308			
59	SI3~ccs	0.19335524	0.166189	1.16346596	0.2446405	-0.1323692 to 0.51907969			
60	ED~LPS	0.00731068	0.02370251	0.30843479	0.75775151	-0.0391454 to 0.05376675			
61	ED~Age	-0.0410991	0.03294615	-1.2474622	0.21222809	-0.1056724 to 0.02347419			
62	ED~Sex	0.03432647	0.0652855	0.52579001	0.59903411	-0.0936308 to 0.1622837			
63	ED~Feeds	-0.1072598	0.07828966	-1.3700383	0.17067493	-0.2607048 to 0.04618508			
64	ED~nutritional	0.09338917	0.03940011	2.37027691	0.01777477	0.01616637 to 0.17061197			
65	ED~hhc	0.00894906	0.01436387	0.62302589	0.53326751	-0.0192036 to 0.03710174			
66	ED~underlying	-0.0221932	0.05027163	-0.4414653	0.65887621	-0.1207238 to 0.0763374			
67	ED~acute	0.0761719	0.03808642	1.99997516	0.04550295	0.00152388 to 0.15081992			
68	ED~ccs	0.19596834	0.18065765	1.08474976	0.2780325	-0.1581141 to 0.55005082			
69	LPS~Age	0.00508635	0.06104153	0.08332605	0.93359228	-0.1145528 to 0.12472554			
70	LPS~Sex	-0.0071103	0.12305987	-0.057779	0.95392468	-0.2483032 to 0.23408264			
71	LPS~Feeds	0.02957432	0.14388546	0.20554073	0.83714968	-0.252436 to 0.31158464			
72	LPS~nutritional	0.06471792	0.07300297	0.88651073	0.37534238	-0.0783653 to 0.20780112			
73	LPS~hhc	0.04123624	0.02670447	1.54417004	0.12254714	-0.0111036 to 0.09357603			
74	LPS~underlying	0.04223963	0.09706739	0.43515782	0.66344788	-0.1480089 to 0.23248822			
75	LPS~acute	0.01304635	0.07244064	0.18009707	0.85707637	-0.1289347 to 0.15502739			
76	LPS~ccs	0.3722121	0.33198535	1.12117024	0.26221541	-0.2784672 to 1.02289143			
77	WAD at Discharge~GM1	-0.2573212	0.03553712	-7.240911	4.46E-13	-0.3269726 to -0.1876697			
78	WAD at Discharge~GM2	-0.1803775	0.04519873	-3.9907646	6.59E-05	-0.2689653 to -0.0917896			
79	WAD at Discharge~SI1	-0.0388007	0.03491212	-1.1113828	0.26640361	-0.1072272 to 0.02962576			
80	WAD at Discharge~SI2	-0.0472952	0.04014114	-1.1782223	0.23870799	-0.1259704 to 0.03138			
81	WAD at Discharge~SI3	0.05771588	0.05015494	1.15075158	0.24983445	-0.040586 to 0.15601775			

82	WAD at Discharge~ED	-0.0124454	0.08047899	-0.1546421	0.87710348	-0.1701814 to 0.14529048			
83	WAD at Discharge~LPS	0.00999783	0.02574351	0.388363	0.69774742	-0.0404585 to 0.06045418			
84	WAD at Discharge~Age	0.3124248	0.03783498	8.25756498	2.22E-16	0.2382696 to 0.38657999			
85	WAD at Discharge~Sex	0.04835052	0.07167193	0.6746088	0.49992434	-0.0921239 to 0.18882492			
86	WAD at Discharge~Feeds	0.75185976	0.08492164	8.85357057	0	0.5854164 to 0.91830312			
87	WAD at Discharge~nutritional	0.18746264	0.04299131	4.36047749	1.30E-05	0.10320122 to 0.27172407			
88	WAD at Discharge~hhc	-0.0193371	0.01558827	-1.2404897	0.2147943	-0.0498895 to 0.01121536			
89	WAD at Discharge~underlying	0.95741701	0.05703266	16.7871717	0	0.84563505 to 1.06919896			
90	WAD at Discharge~ccs	0.28050223	0.1954269	1.43533069	0.15119289	-0.1025275 to 0.66353192			
91	WAD at Discharge~acute	0.00414537	0.04292879	0.0965638	0.9230728	-0.0799935 to 0.08828424			
92	Delta_WAD_fu90~WAD at Discharge	0.07065374	0.03544057	1.99358371	0.04619757	0.0011915 to 0.14011598			
93	Delta_WAD_fu90~GM1	-0.1336294	0.03046467	-4.3863728	1.15E-05	-0.1933391 to -0.0739197			
94	Delta_WAD_fu90~GM2	-0.0876393	0.03827516	-2.2897178	0.02203768	-0.1626573 to -0.0126214			
95	Delta_WAD_fu90~SI1	-0.0530605	0.02912012	-1.8221252	0.06843599	-0.1101349 to 0.00401388			
96	Delta_WAD_fu90~SI2	0.05188365	0.03349455	1.54901736	0.12137755	-0.0137645 to 0.11753177			
97	Delta_WAD_fu90~SI3	0.00394339	0.04066884	0.09696342	0.92275545	-0.0757661 to 0.08365285			
98	Delta_WAD_fu90~ED	0.00419493	0.06376637	0.06578591	0.94754827	-0.1207849 to 0.12917473			
99	Delta_WAD_fu90~LPS	0.0117612	0.02153559	0.54612836	0.5849777	-0.0304478 to 0.05397019			
100	Delta_WAD_fu90~Age	0.02229472	0.03323831	0.67075364	0.50237748	-0.0428512 to 0.0874406			
101	Delta_WAD_fu90~Sex	-0.0780272	0.05850832	-1.3336088	0.1823321	-0.1927014 to 0.03664699			
102	Delta_WAD_fu90~Feeds	0.10901857	0.07363864	1.48045318	0.13875235	-0.0353105 to 0.25334766			
103	Delta_WAD_fu90~nutritional	0.0604647	0.03620134	1.67023381	0.09487311	-0.0104886 to 0.13141802			
104	Delta_WAD_fu90~hhc	-0.0122071	0.01265677	-0.964468	0.33481136	-0.0370139 to 0.01259977			
105	Delta_WAD_fu90~underlying	-0.0468123	0.05654696	-0.8278474	0.4077569	-0.1576423 to 0.06401775			
106	Delta_WAD_fu90~ccs	-0.0442542	0.16100574	-0.2748609	0.78342314	-0.3598196 to 0.27131128			
107	Delta_WAD_fu90~acute	-0.0116103	0.03431063	-0.3383877	0.73507102	-0.0788579 to 0.05563731			
108	CAL~~CAL	0.14463592	0.02863529	5.05096815	4.40E-07	0.08851179 to 0.20076004			
109	MPO~~MPO	0.2731454	0.03251421	8.40079997	0	0.20941872 to 0.33687209			
110	AAT~~AAT	0.31371178	0.02515973	12.4688049	0	0.26439962 to 0.36302395			
111	GM1~~GM1	0.94891842	0.05738876	16.5349181	0	0.83643852 to 1.06139832			
112	GM2~~GM2	0.58537638	0.0353311	16.5683012	0	0.51612868 to 0.65462407			
113	SI1~~SI1	1.04611661	0.06554145	15.9611449	0	0.91765773 to 1.1745755			
114	SI2~~SI2	0.74172742	0.04503284	16.4708122	0	0.65346468 to 0.82999016			
115	SI3~~SI3	0.48797547	0.0294756	16.5552347	0	0.43020435 to 0.54574658			
116	LPS~~LPS	1.90197283	0.11663022	16.3077184	0	1.67338179 to 2.13056386			
117	WAD at Discharge~~WAD at Discharge	0.65251249	0.03935033	16.5821343	0	0.57538725 to 0.72963772			
118	Delta_WAD_fu90~~Delta_WAD_fu90	0.38162625	0.02438256	15.6516045	0	0.33383731 to 0.4294152			
119	ED~~ED	0.33422959	0.04263806	7.83876247	4.44E-15	0.25066054 to 0.41779865			
120	Age~~Age	0.99818182	0	NA	NA	0.99818182 to 0.99818182			
121	Age~~Sex	0.02229946	0	NA	NA	0.02229946 to 0.02229946			
122	Age~~Feeds	0.02212228	0	NA	NA	0.02212228 to 0.02212228			
123	Age~~nutritional	0.14534638	0	NA	NA	0.14534638 to 0.14534638			

124	Age~~hhc	0.20747632	0	NA	NA	0.20747632 to 0.20747632			
125	Age~~underlying	0.0570261	0	NA	NA	0.0570261 to 0.0570261			
126	Age~~acute	-0.0610653	0	NA	NA	-0.0610653 to -0.0610653			
127	Age~~ccs	0.00455744	0	NA	NA	0.00455744 to 0.00455744			
128	Sex~~Sex	0.24106116	0	NA	NA	0.24106116 to 0.24106116			
129	Sex~~Feeds	0.00656198	0	NA	NA	0.00656198 to 0.00656198			
130	Sex~~nutritional	-0.0050728	0	NA	NA	-0.0050728 to -0.0050728			
131	Sex~~hhc	-0.0626071	0	NA	NA	-0.0626071 to -0.0626071			
132	Sex~~underlying	0.03957197	0	NA	NA	0.03957197 to 0.03957197			
133	Sex~~acute	-0.0296124	0	NA	NA	-0.0296124 to -0.0296124			
134	Sex~~ccs	-0.0014147	0	NA	NA	-0.0014147 to -0.0014147			
135	Feeds~~Feeds	0.24867769	0	NA	NA	0.24867769 to 0.24867769			
136	Feeds~~nutritional	0.14920359	0	NA	NA	0.14920359 to 0.14920359			
137	Feeds~~hhc	-0.2616987	0	NA	NA	-0.2616987 to -0.2616987			
138	Feeds~~underlying	0.14004145	0	NA	NA	0.14004145 to 0.14004145			
139	Feeds~~acute	-0.045948	0	NA	NA	-0.045948 to -0.045948			
140	Feeds~~ccs	-0.0023728	0	NA	NA	-0.0023728 to -0.0023728			
141	nutritional~~nutritional	0.82449123	0	NA	NA	0.82449123 to 0.82449123			
142	nutritional~~hhc	0.14317948	0	NA	NA	0.14317948 to 0.14317948			
143	nutritional~~underlying	0.13408045	0	NA	NA	0.13408045 to 0.13408045			
144	nutritional~~acute	-0.1076986	0	NA	NA	-0.1076986 to -0.1076986			
145	nutritional~~ccs	0.01829404	0	NA	NA	0.01829404 to 0.01829404			
146	hhc~~hhc	5.79472351	0	NA	NA	5.79472351 to 5.79472351			
147	hhc~~underlying	0.00801339	0	NA	NA	0.00801339 to 0.00801339			
148	hhc~~acute	-0.0604445	0	NA	NA	-0.0604445 to -0.0604445			
149	hhc~~ccs	0.09311814	0	NA	NA	0.09311814 to 0.09311814			
150	underlying~~underlying	0.47441845	0	NA	NA	0.47441845 to 0.47441845			
151	underlying~~acute	-0.0329238	0	NA	NA	-0.0329238 to -0.0329238			
152	underlying~~ccs	0.00275896	0	NA	NA	0.00275896 to 0.00275896			
153	acute~~acute	0.7072059	0	NA	NA	0.7072059 to 0.7072059			
154	acute~~ccs	0.01113828	0	NA	NA	0.01113828 to 0.01113828			
155	ccs~~ccs	0.03455135	0	NA	NA	0.03455135 to 0.03455135			
156	CAL~1	2.53419363	0.17149409	14.7771484	0	2.19807139 to 2.87031588			
157	MPO~1	3.02853575	0.17461334	17.3442403	0	2.68629989 to 3.3707716			
158	AAT~1	2.84103124	0.11722828	24.2350329	0	2.61126803 to 3.07079445			
159	GM1~1	0.28732934	0.22451903	1.27975492	0.20063134	-0.1527199 to 0.72737855			
160	GM2~1	0.51935848	0.17608999	2.94939244	0.00318399	0.17422844 to 0.86448852			
161	SI1~1	0.2770144	0.24037003	1.15244981	0.24913628	-0.1941022 to 0.748131			
162	SI2~1	0.29511707	0.19847247	1.48694212	0.1370301	-0.0938818 to 0.68411596			
163	SI3~1	0.07031064	0.16031152	0.43858756	0.66096041	-0.2438942 to 0.38451544			
164	LPS~1	2.19670219	0.3082759	7.12576678	1.03E-12	1.59249252 to 2.80091186			
165	WAD at Discharge~1	0.95331222	0.18729988	5.08976412	3.59E-07	0.5862112 to 1.32041324			
166	Delta_WAD_fu90~1	0.02056997	0.15627709	0.13162501	0.8952809	-0.2857275 to 0.32686743			
167	Age~1	-2.12E-15	0	NA	NA	-2.12E-15 to -2.12E-15			
168	Sex~1	1.59454545	0	NA	NA	1.59454545 to 1.59454545			
169	Feeds~1	1.53636364	0	NA	NA	1.53636364 to 1.53636364			

170	nutritional~1	-0.0693206	0	NA	NA	-0.0693206 to -0.0693206			
171	hhc~1	-0.0161151	0	NA	NA	-0.0161151 to -0.0161151			
172	underlying~1	-0.0392955	0	NA	NA	-0.0392955 to -0.0392955			
173	acut~1	-0.0675408	0	NA	NA	-0.0675408 to -0.0675408			
174	ccs~1	-0.0050275	0	NA	NA	-0.0050275 to -0.0050275			
175	ED~1	0	0	NA	NA	0 to 0			

Regression analysis from a structural equation model (SEM) examining the relationships between the first three components of both systemic inflammation and growth mediators and growth and how they relate to basal WAD at discharge, enteric inflammation and permeability, receipt of therapeutic and socio-economic, demographic and medical factors. Estimate represents standardized estimates of the individual relationships within the SEM resulting from simple linear regressions. Estimates and p-values were obtained using the sem function within the lavaan (version 0.6.17) package in R and associations with $p<0.05$ were considered statistically significant. The overall model fit indices were chi-square ($p=0.016$), comparative fit index (CFI; 0.985), root mean square error for approximation (RMSEA; 0.032) and standardised root mean squared residual (SRMR; 0.023) and confirmed model adequacy. ED = Enteric Dysfunction, CAL = Calprotectin, MPO = Myeloperoxidase, AAT = Alpha-1-Antitrypsin, LPS = Lipopolysaccharides, GM1 = Growth Mediator Component 1, GM2 = Growth Mediator Component 2, SI1 = systemic inflammation Component 1, SI2 = systemic inflammation Component 2, SI3 = systemic inflammation Component 3, Feeds = receipt of therapeutic feeds, nutritional = age-inappropriate nutrition, hhc = household-level exposures, underlying = underlying chronic conditions, acute = clinical presentation, ccs = caregiver characteristics, WAD = Weight Absolute Deficit, and Delta_WAD_fu90 = Delta-WAD at 3m post-discharge. ~ = path, ~~ = covariance, NA = Not Applicable. npar = number of free (estimated) parameters. fmin - minimized fit function value (model–data discrepancy; not usually compared across datasets). logl, unrestricted.logl = log-likelihood of your model and of the saturated (unrestricted) model. aic, bic, bic2 = information criteria. ntotal = total N. Chisq: The model Chi-squared assesses overall fit and the discrepancy between the sample and fitted covariance matrices. Its p-value should be > .05 (i.e., the hypothesis of a perfect fit cannot be rejected). However, it is quite sensitive to sample size. gfi/agfi: The (Adjusted) Goodness of Fit is the proportion of variance accounted for by the estimated population covariance. The gfi and the agfi should be > .95 and > .90, respectively. pgfi = Parsimonious GFI (penalized). mfi = McDonald’s Fit Index; higher better (1 = perfect). ecvi = Expected Cross-Validation Index; lower suggests better out-of-sample fit (compare across models on same data). nfi/nnfi/tli: The (Non) Normed Fit Index. An NFI of 0.95, indicates the model of interest improves the fit by 95\ null model. The NNFI (also called the Tucker Lewis index; TLI) is preferable for smaller samples. They should be > .90 or > .95. CFI = The Comparative Fit Index is a revised form of NFI. Not very sensitive to sample size. Compares the fit of a target model to the fit of an independent, or null, model. It should be > .90. RMSEA = The Root Mean Square Error of Approximation is a parsimony-adjusted index. Values closer to 0 represent a good fit. It should be < .08 or < .05. The p-value printed with it tests the hypothesis that RMSEA is less than or equal to .05 (a cutoff sometimes used for good fit), and thus should be not significant. rmsea.ci.lower, rmsea.ci.upper, rmsea.ci.level = RMSEA CI bounds and confidence level. RMR/SRMR: the (Standardized) Root Mean Square Residual represents the square-root of the difference between the residuals of the sample covariance matrix and the hypothesized model. As the RMR can be sometimes hard to interpret, better to use SRMR. Should be < .08. RFI = the Relative Fit Index, also known as RHO1, is not guaranteed to vary from 0 to 1. However, RFI close to 1 indicates a good fit. IFI = the Incremental Fit Index (IFI) adjusts the Normed Fit Index (NFI) for sample size and degrees of freedom. Over 0.90 is a good fit, but the index can exceed 1.
