# Supplementary material for: Inflammation impairs post-hospital discharge growth among children hospitalised with acute illness in sub-Saharan Africa and south Asia
Source: Nat Commun. 2025 Nov 28;16:10788. doi: 10.1038/s41467-025-66245-2 (PMC12663110; doi:10.1038/s41467-025-66245-2)
Supplement: Supplementary file 3 — Reporting Summary [file 41467_2025_66245_MOESM3_ESM.pdf]

nature portfolio

Corresponding author(s): James M. Njunge

Last updated by author(s): 2025/05/03

Reporting Summary

Nature Portfolio wishes to improve the reproducibility of the work that we publish. This form provides structure for consistency and transparency in reporting. For further information on Nature Portfolio policies, see our Editorial Policies and the Editorial Policy Checklist.

Statistics

For all statistical analyses, confirm that the following items are present in the figure legend, table legend, main text, or Methods section.

n/a	Confirmed	
☐	☒	The exact sample size (*n*) for each experimental group/condition, given as a discrete number and unit of measurement
☐	☒	A statement on whether measurements were taken from distinct samples or whether the same sample was measured repeatedly
☐	☒	The statistical test(s) used AND whether they are one- or two-sided<br>*Only common tests should be described solely by name; describe more complex techniques in the Methods section.*
☐	☒	A description of all covariates tested
☐	☒	A description of any assumptions or corrections, such as tests of normality and adjustment for multiple comparisons
☐	☒	A full description of the statistical parameters including central tendency (e.g. means) or other basic estimates (e.g. regression coefficient) AND variation (e.g. standard deviation) or associated estimates of uncertainty (e.g. confidence intervals)
☐	☒	For null hypothesis testing, the test statistic (e.g. *F*, *t*, *r*) with confidence intervals, effect sizes, degrees of freedom and *P* value noted<br>*Give P values as exact values whenever suitable.*
☒	☐	For Bayesian analysis, information on the choice of priors and Markov chain Monte Carlo settings
☐	☒	For hierarchical and complex designs, identification of the appropriate level for tests and full reporting of outcomes
☒	☐	Estimates of effect sizes (e.g. Cohen's *d*, Pearson's *r*), indicating how they were calculated

Our web collection on statistics for biologists contains articles on many of the points above.

Software and code

Policy information about availability of computer code

Data collection

The current study is a retrospective study nested within a larger cohort study. Therefore, no data collection was done.

Data analysis

R version 4.4.2 and R studio Version 2024.09.1+394 (2024.09.1+394) was used for all data analysis. The codes used in this study are hosted on the Harvard Dataverse website under https://doi.org/10.7910/DVN/TBQYSF.
Below are the packages and respective versions:
library(arsenal) # version 3.6.3
library(Boruta) # version 8.0.0
library(broom) # version 1.0.7
library(cardx) # version 0.2.2
library(caret) # version 6.0-94
library(circlize) # version 0.4.16
library(cli) # version 3.6.3
library(corrgram) # version 1.14
library(corrplot) # version 0.95
library(cowplot) # version 1.1.3
library(data.table) # version 1.16.2
library(DataExplorer) # version 0.8.3
library(datawizard) # version 0.13.0
library(dendextend) # version 1.18.1
library(DiagrammeRsvg) # version 0.1
library(dplyr) # version 1.1.4

```
library(DT) # version 0.33
library(extrafont) # version 0.19
library(factoextra) # version 1.0.7
library(FactoMineR) # version  2.11
library(forcats) # version 1.0.0
library(foreign) # version 0.8-87
library(ggbeeswarm) # version 0.7.2
library(ggcorrplot) # version   0.1.4.1
library(ggfortify) # version 0.4.17
library(ggplot2) # version 3.5.1
library(ggpubr) # version 0.6.0
library(ggsci) # version 3.2.0
library(ggstatsplot) # version 0.12.4
library(ggthemes) # version 5.1.0
library(glmnet) # version 4.1-8
library(GPArotation) # version  2024.3-1
library(gtsummary) # version 2.0.3
library(haven) # version 2.5.4
library(here) # version 1.0.1
library(Hmisc) # version 5.1-3
library(kableExtra) # version 1.4.0
library(knitr) # version  1.48
library(lattice) # version 0.22-6
library(lavaan) # version 0.6-19
library(lavaanPlot) # version 0.8.1
library(lcmm) # version 2.1.0
library(lme4) # version  1.1-35.5
library(lmerTest) # version 3.1-3
library(MASS) # version 7.3-61
library(MatchIt) # version 4.5.5
library(mgsub) # version 1.7.3
library(mlbench) # version 2.1-5
library(multcomp) # version 1.4-26
library(naniar) # version 1.1.0
library(nFactors) # version  2.4.1.1
library(OlinkAnalyze) # version 4.0.1
library(openxlsx) # version   4.2.7.1
library(outliers) # version  0.15
library(pacman) # version 0.5.1
library(PerformanceAnalytics) # version 2.0.4
library(plotly) # version 4.10.4
library(PMCMRplus) # version 1.9.12
library(psych) # version  2.4.6.26
library(qwraps2) # version 0.6.1
library(randomForest) # version   4.7-1.2
library(RColorBrewer) # version 1.1-3
library(readr) # version 2.1.5
library(readxl) # version 1.4.3
library(reshape) # version 0.8.9
library(reshape2) # version 1.4.4
library(rlang) # version 1.1.4
library(ROCR) # version 1.0-11
library(rstatix) # version 0.7.2
library(rsvg) # version 2.6.1
library(scales) # version 1.3.0
library(stringr) # version 1.5.1
library(tibble) # version 3.2.1
library(tidyverse) # version 2.0.0
library(tinytex) # version  0.53
library(visdat) # version 0.6.0
```

For manuscripts utilizing custom algorithms or software that are central to the research but not yet described in published literature, software must be made available to editors and reviewers. We strongly encourage code deposition in a community repository (e.g. GitHub). See the Nature Portfolio guidelines for submitting code & software for further information.

Data

Policy information about availability of data

All manuscripts must include a data availability statement. This statement should provide the following information, where applicable:

- Accession codes, unique identifiers, or web links for publicly available datasets
- A description of any restrictions on data availability
- For clinical datasets or third party data, please ensure that the statement adheres to our policy

The data that support the findings of this study are archived on the Harvard Dataverse (https://doi.org/10.7910/DVN/TBQYSF). The data contain sensitive information about study participants and may include identifiers that could compromise confidentiality or lead to ethnic stigmatisation. Access to these data requires submission of a formal request for consideration by our Data Governance Committee. Email completed data request form to the Data Governance Committee at dgc@kemri-wellcome.org.The requester provides investigators details, variables requested, intended use of the dataset, potential risks of the study including risks to confidentiality of individuals or communities, potential benefits of the study including to participant communities, scientific capacity building or health policy and planned outputs (if analysis on dataset will result in publication or reports or presentations). The requester also needs to formally agree to the conditions and limitations for data sharing to avoid misuse of shared data.

Research involving human participants, their data, or biological material

Policy information about studies with human participants or human data. See also policy information about sex, gender (identity/presentation), and sexual orientation and race, ethnicity and racism.

Reporting on sex and gender	The analysis considered sex as a biological attribute of the child at birth as male and female. Sex was adjusted in all models where relevant. We have also provided the analysis stratified by sex.
Reporting on race, ethnicity, or other socially relevant groupings	This study did not group the participants based on their races or ethnicity. Structural equation modelling (SEM) path models were used to examine how adverse household and chronic medical conditions, enteric inflammation and permeability, systemic inflammation, and growth mediators influence weight gain. The household and chronic medical conditions were latent variables built to represent key domains of exposures which were designed to reflect the UNICEF conceptual framework. Domains were validated using confirmatory factor analysis. The exposure domains were latent variables constructed to summarise correlated elements into a score attributed to each child (e.g., age-inappropriate nutrition is constructed from 3 variables: recommended appropriate diet, poor feeding, and recent weight loss. Household-level exposures is constructed from 4 variables- Low household assets; low food security; unimproved toilet; poor water availability and classified as least-, moderate-, most- adverse). These latent scores were split into tertiles, and a child classified within the “high” or “most-adverse” tertile indicates higher risk with regards to the exposure domain. The final SEM models included the biological factors, demographic factors comprising age, site and sex, receipt of therapeutic feeds as well as latent variables depicting socioeconomic and medical factors. Because of the potential site specific effects, site was included in the regression models as a random effect.
Population characteristics	The CHAIN cohort was conducted at nine hospitals in Africa and South Asia: Dhaka and Matlab Hospitals (Bangladesh), Banfora Referral Hospital (Burkina Faso), Kilifi County, Mbagathi County and Migori County Hospitals (Kenya), Queen Elizabeth Hospital (Malawi), Civil Hospital (Pakistan), and Mulago National Referral Hospital (Uganda). The Banfora, Dhaka and Kampala sites had larger proportions of study children compared to the other study sites. Selected children were mainly diagnosed with pneumonia and diarrhoea at admission and the proportion of non-wasted to severely wasted was similar. Most haematological parameters were comparable by sex except eosinophils which were increased among males at discharge. Several parameters varied by nutritional status; albumin and erythrocytes were lower while white blood cells, platelets, neutrophils, and monocytes counts were higher among severely wasted children. Males were more underweight and stunted and had larger weight deficits at discharge and at 3 months post discharge. However, the proportion of the not wasted, moderately wasted and the severely wasted did not vary by sex.
Recruitment	The primary study, CHAIN, recruited acutely ill children aged 2-23 months stratified by anthropometry using mid-upper-arm circumference (MUAC) into: no wasting (MUAC ≥12·5 cm [age ≥6 months] or MUAC≥12·0 cm [age <6 months]), moderate wasting (MUAC 11·5–12·5 cm [age ≥6 months] or MUAC 11·0–12·0 cm [age <6 months]), and severe wasting (MUAC <11·5 cm [age ≥6 months] or MUAC <11·0 cm [age <6 months], or bilateral pedal oedema [kwashiorkor] unexplained by other medical causes) at hospital admission.
Ethics oversight	Oxford Tropical Research Ethics Committee, UK<br>The Kenya Medical Research Institute, Kenya<br>Makerere University School of Biomedical Sciences Research Ethics Committee and The Uganda National Council for Science and Technology, Uganda<br>Aga Khan University, Pakistan<br>International Centre for Diarrhoeal Disease Research, (icddr,b), Bangladesh<br>The University of Malawi<br>The University of Ouagadougou and Centre Muraz, Burkina Faso

Note that full information on the approval of the study protocol must also be provided in the manuscript.

Field-specific reporting

Please select the one below that is the best fit for your research. If you are not sure, read the appropriate sections before making your selection.

☒ Life sciences ☐ Behavioural & social sciences ☐ Ecological, evolutionary & environmental sciences

For a reference copy of the document with all sections, see nature.com/documents/nr-reporting-summary-flat.pdf

Life sciences study design

All studies must disclose on these points even when the disclosure is negative.

Sample size	No formal sample size calculation was performed for this secondary analysis study. A total of 550 children being discharged from hospital randomly selected from the CHAIN study were included for analysis.
Data exclusions	We excluded deaths, children with oedema and those missing or insufficient samples. Children with missing samples at discharge generally had better anthropometric indices than those with analysed samples and Blantyre and Karachi had more children with missing samples compared to the other sites. There is likely selection and attrition bias at discharge due to exclusion of children who lacked or had insufficient samples, deaths, had nutritional oedema and those lost to follow-up (loss to follow-up within the CHAIN study cohort was low; 3.7%).
Replication	The results can be reproduced since data and codes are available. The replication will be conducted with future datasets.
Randomization	Data used in this analysis was generated from children who had been randomly sampled at 24% using a random number generator from the original cohort.
Blinding	Since this is a retrospective study, blinding as not possible for growth analysis.

Reporting for specific materials, systems and methods

We require information from authors about some types of materials, experimental systems and methods used in many studies. Here, indicate whether each material, system or method listed is relevant to your study. If you are not sure if a list item applies to your research, read the appropriate section before selecting a response.

Materials & experimental systems

n/a	Involved in the study	
☒	☐	Antibodies
☒	☐	Eukaryotic cell lines
☒	☐	Palaeontology and archaeology
☒	☐	Animals and other organisms
☒	☐	Clinical data
☒	☐	Dual use research of concern
☒	☐	Plants

Methods

n/a	Involved in the study	
☒	☐	ChIP-seq
☒	☐	Flow cytometry
☒	☐	MRI-based neuroimaging

Plants

Seed stocks	*Report on the source of all seed stocks or other plant material used. If applicable, state the seed stock centre and catalogue number. If plant specimens were collected from the field, describe the collection location, date and sampling procedures.*
Novel plant genotypes	*Describe the methods by which all novel plant genotypes were produced. This includes those generated by transgenic approaches, gene editing, chemical/radiation-based mutagenesis and hybridization. For transgenic lines, describe the transformation method, the number of independent lines analyzed and the generation upon which experiments were performed. For gene-edited lines, describe the editor used, the endogenous sequence targeted for editing, the targeting guide RNA sequence (if applicable) and how the editor was applied.*
Authentication	*Describe any authentication procedures for each seed stock used or novel genotype generated. Describe any experiments used to assess the effect of a mutation and, where applicable, how potential secondary effects (e.g. second site T-DNA insertions, mosiacism, off-target gene editing) were examined.*
